# Dynamic SUMOylation of MORC2 orchestrates chromatin remodelling and DNA repair in response to DNA damage and drives chemoresistance in breast cancer

**DOI:** 10.7150/thno.79688

**Published:** 2023-01-22

**Authors:** Fang-Lin Zhang, Shao-Ying Yang, Li Liao, Tai-Mei Zhang, Yin-Ling Zhang, Shu-Yuan Hu, Ling Deng, Min-Ying Huang, Lisa Andriani, Xiao-Yan Ma, Zhi-Min Shao, Da-Qiang Li

**Affiliations:** 1 Fudan University Shanghai Cancer Center and Institutes of Biomedical Sciences, Shanghai Medical College, Fudan University, Shanghai 200032, China; 2 Cancer Institute, Shanghai Medical College, Fudan University, Shanghai 200032, China; 3 Department of Oncology, Shanghai Medical College, Fudan University, Shanghai 200032, China; 4 Department of Breast Surgery, Shanghai Medical College, Fudan University, Shanghai 200032, China; 5 Shanghai Key Laboratory of Breast Cancer, Shanghai Medical College, Fudan University, Shanghai 200032, China; 6 Shanghai Key Laboratory of Radiation Oncology, Shanghai Medical College, Fudan University, Shanghai, China

**Keywords:** Breast cancer, MORC2, SUMOylation, DNA damage response, Therapeutic resistance

## Abstract

**Rationale:** SUMOylation regulates a plethora of biological processes, and its inhibitors are currently under investigation in clinical trials as anticancer agents. Thus, identifying new targets with site-specific SUMOylation and defining their biological functions will not only provide new mechanistic insights into the SUMOylation signaling but also open an avenue for developing new strategy for cancer therapy. MORC family CW-type zinc finger 2 (MORC2) is a newly identified chromatin-remodeling enzyme with an emerging role in the DNA damage response (DDR), but its regulatory mechanism remains enigmatic.

**Methods:** *In vivo* and *in vitro* SUMOylation assays were used to determine the SUMOylation levels of MORC2. Overexpression and knockdown of SUMO-associated enzymes were used to detect their effects on MORC2 SUMOylation. The effect of dynamic MORC2 SUMOylation on the sensitivity of breast cancer cells to chemotherapeutic drugs was examined through *in vitro* and *in vivo* functional assays. Immunoprecipitation, GST pull-down, MNase, and chromatin segregation assays were used to explore the underlying mechanisms.

**Results:** Here, we report that MORC2 is modified by small ubiquitin-like modifier 1 (SUMO1) and SUMO2/3 at lysine 767 (K767) in a SUMO-interacting motif dependent manner. MORC2 SUMOylation is induced by SUMO E3 ligase tripartite motif containing 28 (TRIM28) and reversed by deSUMOylase sentrin-specific protease 1 (SENP1). Intriguingly, SUMOylation of MORC2 is decreased at the early stage of DNA damage induced by chemotherapeutic drugs that attenuate the interaction of MORC2 with TRIM28. MORC2 deSUMOylation induces transient chromatin relaxation to enable efficient DNA repair. At the relatively late stage of DNA damage, MORC2 SUMOylation is restored, and SUMOylated MORC2 interacts with protein kinase CSK21 (casein kinase II subunit alpha), which in turn phosphorylates DNA-PKcs (DNA-dependent protein kinase catalytic subunit), thus promoting DNA repair. Notably, expression of a SUMOylation-deficient mutant MORC2 or administration of SUMO inhibitor enhances the sensitivity of breast cancer cells to DNA-damaging chemotherapeutic drugs.

**Conclusions:** Collectively, these findings uncover a novel regulatory mechanism of MORC2 by SUMOylation and reveal the intricate dynamics of MORC2 SUMOylation important for proper DDR. We also propose a promising strategy to sensitize MORC2-driven breast tumors to chemotherapeutic drugs by inhibition of the SUMO pathway.

## Introduction

Genomic DNA is constantly assaulted by endogenous and exogeneous DNA-damaging agents that induce DNA damage. To counter this threat, cells have evolved a coordinated signaling network, collectively termed the DNA-damage response (DDR), for detection, signaling, and repair of DNA lesions in the context of chromatin 1. Naturally, the condensed chromatin structure imposes a major constraint on the DDR as it impedes the accessibility of DNA repair factors to damaged DNA 2. To enable DNA repair, chromatin undergoes transient relaxation when DNA damage is introduced, followed by restoration of chromatin architecture once DNA repair is complete 3-5. Dynamic change of chromatin structure during the DDR has been integrated into the access/prime-repair-restore model 6, 7. However, how this event is regulated is still not well understood.

The DDR entails spatiotemporal regulation of posttranslational modifications (PTMs) of chromatin-associated proteins 8, 9. One of the most prevalent PTMs is SUMOylation, which involves the covalent attachment of small-ubiquitin-like modifier (SUMO) to lysine residues on substrates 10. In mammals, three major SUMO isoforms have been identified, including SUMO1, SUMO2, and SUMO3. Human SUMO2 and SUMO3 share 97% sequence identity (collectively referred to as SUMO2/3) and form poly-SUMO chains 11. In contrast, SUMO1 exhibits 46% amino acid sequence identity with SUMO2/3, and usually modifies a substrate as a monomer 11, 12. Of note, some substrates can be exclusively modified by SUMO1 or SUMO2/3, whereas others can be modified by all of three SUMO isoforms 10, 13.

Similar to ubiquitination, SUMOylation is catalyzed by a cascade of enzymes initiated by E1 SUMO-activating enzymes (SAE1/SAE2), a single E2 conjugating enzyme (UBC9), and a limited set of E3 SUMO ligases 10. De-SUMOylation is carried out by several sentrin-specific proteases (SENPs) that remove SUMO from modified substrates 14. In addition to covalent conjugation to substrates, SUMOs can interact with other proteins harboring one or more functional SUMO-interaction motifs (SIMs) to assemble larger protein complexes 12. The SIM is also required for efficient SUMOylation of some SUMO substrates, such as oncoprotein MTA1 15 and transcriptional corepressor DAXX 16.

SUMOylation has been shown to affect subcellular localization, stability, or activity of target proteins, thus regulating a plethora of biological processes 10. In response to cellular or environmental stress, the dynamic balance between SUMOylation and deSUMOylation of DDR factors is critical for proper DDR 17, 18. Moreover, selective inhibitors of SUMOylation have been shown to exert potent anticancer effects as a single agent or in combination with other anti-cancer agents in preclinical model systems 19, 20. Thus, identifying new targets with site-specific SUMOylation and defining their biological functions will not only provide new mechanistic insights into the implication of the SUMO signaling in the DDR but also open an avenue for developing new strategy for cancer therapy 17.

Accumulating evidence shows that chromatin-modifying enzymes play a central role in the alteration of chromatin structure and efficient DNA repair after DNA damage 21. Microrchidia CW-type zinc finger 2 (MORC2) is an ATPase-dependent chromatin-remodeling enzyme, which contains a GHKL-type ATPase domain, a CW-type zinc finger (ZF-CW) domain, a chromo-like domain, and three coiled-coil domains 22, 23. Recent studies revealed that MORC2 contributes to epigenetic gene silencing and heterochromatin formation through interaction with the human silencing hub (HUSH) complex 24-27. In addition, MORC2 has an emerging role in the DNA damage response 23, 28-30. Consequently, dysregulation of MORC2 has been linked with the development of multiple types of human cancer and several genetic disorders 24, 31-34. Interestingly, a recent study documented that MORC2 regulates cell differentiation of mouse myoblasts and human gastric cancer cells through enhancing SUMOylation of transcription factor C/EBPα 35, but whether MORC2 itself is regulated by SUMOylation and its functional consequence remain unknown.

In this study, we provide evidence that MORC2 is modified by SUMO1 and SUMO2/3 at lysine K767 in a SIM-dependent manner, and this event is reversely controlled by the E3 SUMO ligase TRIM28 and the SUMO protease SENP1. In response to DNA-damaging agents, dynamic SUMOylation of MORC2 is important for proper chromatin remodeling and DNA repair. Moreover, expression of a SUMOylation-deficient mutant MORC2 (K767R or SIM mutant) or administration with SUMO inhibitor ML-792 enhances breast cancer cellular sensitivity to DNA-damaging chemotherapeutic drug. Together, these findings provide molecular insights into the mechanism by which SUMOylation controls the function of MORC2 during the DDR process. These results also indicate that targeting MORC2 SUMOylation by small-molecular inhibitors could reverse resistance of breast cancer cells to DNA-damaging chemotherapeutic drugs.

## Materials and methods

### Cell culture

All cell lines were obtained from the Cell Bank of the Type Culture Collection of the Chinese Academy of Sciences (Shanghai, China). They were verified by monitoring mycoplasma contamination and short tandem repeat profiling. Cells were incubated in DMEM or RPMI 1640 media supplemented with 10% (v/v) fetal bovine serum (ExCell Biol, #FSP500), 50 U/mL penicillin and 50 μg/mL streptomycin (BasalMedia, #S110B). The culture conditions were 37 °C under 5% CO_2_ humidified atmosphere. The culture media and supplements were obtained from BasalMedia. ML-792 and doxorubicin (ADR) were purchased from MedChemExpress (# HY-108702) and Selleck Chemicals (#S1208), respectively. The other chemicals were purchased from Sigma-Aldrich unless otherwise specified.

### DNA constructs

Myc-DDK-MORC2 was subcloned into pCDH-CMV-EF1-Puro lentiviral vectors and pLVX-neo-IRES to produce Flag- and HA-tagged expression vectors. Myc-DDK-CBX4, Myc-DDK-TRIM28, and Myc-DDK-UBC9 cDNAs were purchased from Vigene Biosciences, and subcloned into pLVX-neo-IRES vectors to generate HA-CBX4, HA-TRIM28, and HA-UBC9 expression vectors, respectively. GFP-SUMO1/2/3 expression vectors were obtained from Origene (#RG200633, RG224336, and RG200241, respectively). SENP1, SENP2, SENP3, PIAS1, PIAS2a, PIAS2b, PIAS3, and PIAS4 were kindly provided by Dr. Jinke Cheng (Shanghai Jiao Tong University School of Medicine, Shanghai, China). SENP1, SENP2, and SENP3 were subcloned into pLVX-neo-IRES vector. Detailed information of the primers used for molecular cloning is shown in **Table S1.** Short hairpin RNAs (shRNAs) for gene silencing were cloned into pLKO.1 vector (**Table S2**). LentiCas9-Blast (#52962) and lentiGuide-Puro (#52963) were purchased from Addgene. Short guide RNAs (sgRNAs) targeting MORC2, SENP1, and UBC9 were chosen by the Web-based CRISPR design tool analysis from the Zhang lab (http://www.genome-engineering.org/). The sgRNA sequences are listed in **Table S2**. They were cloned into lentiGuide-Puro vector following the standard protocol 36 using a ClonExpress Ultra One Step Cloning kit (Vazyme Biotech, #C115-02). Mutagenesis was performed by PCR using a ClonExpress Ultra One Step Cloning kit and the methylation-sensitive restriction enzyme DpnI (New England Biolabs, #R0176S). All construct sequences were verified by sequencing at Sangon Biotech (Shanghai, China). Detailed information about the primers, including mutagenesis and the truncation constructs, is provided in **Table S3.**

### Plasmid transfection and viral transduction

When cells reached 70% confluence, transient transfections were conducted using DNA transfection reagent (TengyiBio, #TF201201) or LipofectamineTM 2000 (Invitrogen, #11668019) following the manufacturer's instructions. Lentiviral infection was performed as previously described 37. Knockout (KO) cell lines were generated by a CRISPR/Cas9 system 36. Cell lines transcribing shRNA were generated with Vector Backbone pLKO.1-puro. The efficiency of gene silencing was validated by immunoblotting.

### Antibodies, immunoblotting, and immunoprecipitation

Information about antibodies used is listed in **Table S4**. Immunoblotting and immunoprecipitation (IP) analyses were conducted as previously described 37. For immunoblotting, cells were lysed in RIPA buffer (50 mM Tris-HCl, pH7.4, 0.25% sodium deoxycholate, 1 mM EDTA, 150 mM NaCl, 1% NP-40, and 0.1% SDS) supplemented with protease inhibitors (Bimake, #B14002) and phosphatase inhibitors (Bimake, #B15003). Proteins were quantified by bicinchoninic acid assays (Yeasen, #20201ES90), resolved by 6-15% SDS-PAGE, and transferred to polyvinylidene difluoride (PVDF) membranes (EMD Millipore, #IPVH00010). The membranes were incubated with 5% bovine serum albumin (Sigma, #V900933-1KG) for 2 h, and then incubated the indicated antibodies at 4 °C overnight and detected with horseradish peroxidase (HRP)-conjugated rabbit or mouse secondary antibodies (Cell Signaling Technology). The antibody signals were detected with an enhanced chemiluminescence substrate kit (Yeasen, #36208ES80). For IP assays, NP-40 buffer (50 mM Tris-HCl, pH8, 0.5% NP-40, 10% glycerol, 2 mM MgCl_2_, 150 mM NaCl, and 1 mM EDTA) were used for lysis cells at 4 °C, and the lysates were centrifuged for 15 min. The protein extracts were incubated at 4 °C overnight with the listed antibodies and then incubated with protein A/G beads (Bimake, #B23202) or incubated overnight with anti-Flag beads (Shanghai Genomics Technology, #GNI4510-FG). After incubation, the beads were extensively washed to remove nonspecifically bound proteins, dissolved in SDS loading buffer, and analyzed by immunoblotting assays.

### GST pull-down assays

For GST pull-down assays, GST-tagged MORC2 fusion protein was purified using glutathione beads. Recombinant Flag-TRIM28 protein was purchased from Origene (#TP301205). Protein binding was performed in binding buffer (20 mM Tris, pH7.5, 500 mM NaCl, 0.5% NP40, and 10% glycerol) at 4 °C overnight. Pellets were washed three times using washing buffer (10 mM Tris, pH7.5, 150 mM NaCl, and 0.1% NP40), boiled in 2×SDS loading buffer, and then subjected to immunoblotting with the indicated antibodies.

### SUMOylation assays *in vivo* and *in vitro*

For *in vivo* SUMOylation assays, cells with overexpression or knockdown of the indicated genes were lysed in denaturing solution (50 mM Tris-HCl, pH7.4, 300 mM NaCl, 10 mM DTT, 10 mM iodoacetamide, and 1% SDS) supplemented with protease and phosphatase inhibitors, and 10 mM *N*-ethylmaleimide (NEM). The lysates were sonicated and boiled at 100 °C for 5 min and then diluted 10-fold using dilution buffer (150 mM NaCl, 1.7% Thesit, and 50 mM HEPES, pH7.5), followed by centrifuged at 4 °C for 30 min. The lysates were then pulled down using the primary antibodies and protein A/G beads, or anti-Flag beads at 4 °C 15. The pull-down complex was extensively washed. The immunoprecipitated proteins were eluted by boiling in SDS elution sample buffer, separated via SDS-PAGE, and then subjected to immunoblotting assays.

For* in vitro* SUMOylation assays, purified GST-MORC2 fragment (residues 719-1032) containing SUMOylation site K767 from *E. coli* was suspended in reaction buffer containing SUMO enzymes E1 and E2, SUMO molecules SUMO1, SUMO2, and SUMO3, and ATP, with or without SUMO E3 enzyme TRIM28 or deSUMOylase SENP1. After 1 h of incubation at 37 °C, the reactions were terminated by adding 2×SDS loading buffer and were detected by immunoblotting with anti-SUMO1 and anti-SUMO2/3 antibodies.

### Mass spectrometry analysis

Cellular lysates were subjected to IP assays using anti-Flag beads. The immunoprecipitated proteins were washed for three times, eluted by boiling samples in 2× elution buffer, and then subjected to SDS-PAGE. The samples were visualized using Coomassie blue staining and subjected to LC-MS/MS as previously described 37. The data was searched and analyzed according to the Swiss-prot *Homo sapiens* FASTA database (https://www.uniprot.org/proteomes) using the Mascot algorithm. Only peptides with false discovery rate (FDR) < 5% were considered. Proteins with ≥ 2 unique peptides and score > 150 were used for protein identification. All proteins detected in the pCDH control group were considered nonspecific.

### Immunofluorescence and confocal microscopy

Immunofluorescent staining was conducted as described previously 37. Briefly, the cells were placed on coverslips (Thermo Fisher Scientific, #12-545-80) overnight before the treatment. After treatment with DNA-damaging agents, cells were rinsed thrice in ice-cold PBS, fixed with 4% (v/v) formaldehyde (Yeasen, # 36314ES76) for at least 30 min, permeabilized using 0.5% (w/v) Triton X-100 at 4 °C for 10 min, and washed thrice with ice-cold PBS. Cells were then blocked for 30 min at room temperature with 5% (v/v) goat serum and then incubated with primary antibodies (**Table S4**) in 5% BSA at 4 °C overnight. After that, cells were rinsed thrice in PBST and incubated with the appropriate secondary antibodies conjugated with Alexa-568 or Alexa-488 for 1 h. Cells were then washed thrice using PBST and sealed with DAPI-containing Fluoroshield mounting medium (Abcam, #ab104139). Leica SP5 confocal microscope was used for images acquiring through a 63× oil immersion objective lens (Leica Microsystems, Wetzlar, Germany). To analyze the clustered foci, the images were processed and analyzed with the open-source CellProfiler^TM^ software (https://cellprofiler.org/releases) as previously described 38.

### Cell survival assays

For cell viability assays, cells expressing WT or mutant MORC2 were placed onto 96-well plates and incubated overnight. After being treated with the indicated drugs for 72 h, cells were subjected to cell viability assays using a Cell Counting Kit-8 (CCK-8) kit (Yeasen, #40203ES60). The surviving fraction was calculated following a previously report 30. For colony formation assays, cells expressing WT or mutant MORC2 were placed onto 12-well plates in triplicate. After adherence, cells were treated with the indicated drugs for 10-14 days. After being fixed with methanol, survival colonies were visualized by staining with 0.2% crystal violet and counted 23.

### Animal experiments

Animal experiments were conducted following the procedures approved by the Animal Experiments Committee of Fudan University, and according to institutional guidelines for the Care and Use of Laboratory Animals. The stably reconstituted LM2-4175 cells were subcutaneously injected into the flanks of 6-week-old BALB/c female nude mice to generate xenograft tumors. When the tumor volume in one of groups exceeded 100 mm^3^, mice were treated with 3 mg/kg ADR intraperitoneally twice a week. Tumor volume and growth kinetics were measured twice a week and calculated according to the formula of 0.5 × length × width^2^. After the mice were euthanized, the tumors were carefully removed and weighted. Immunohistochemical (IHC) staining of cleaved caspase-3 was performed by Servicebio.

### Micrococcal nuclease (MNase) assays

MNase assays were performed following the protocol as described previously 3, 39. Briefly, isolated nuclei were digested with 10 U/100 µL MNase in digestion buffer (15 mM Tris-HCl, pH7.4, 15 mM NaCl, 60 mM KCl, 1 mM CaCl_2_, 0.25 M sucrose, and 0.5 mM DTT) at 37 °C for 10 min. The digested genomic DNA was carefully purified using a DNA purification kit (Axygen, #AP-PCR-250) and subjected to 1.2% agarose gel electrophoresis.

### Chromatin segregation assays

Chromatin segregation assays were conducted as described previously 40. Briefly, chromatin was extracted by a gradient intensity of successive MNase digestion to produce sequentially enhanced nuclease-resistant nucleosome fractions (C1-C5) which represent varying degrees of accessible chromatin. The fraction with high H3K9me3 (trimethylated histone 3 lysine 9) signaling was sourced mainly from high-order chromatin. DNA damage-induced chromatin relaxation was detected by observing H3K9me3 signaling mobility removed from the C5 fraction.

### Statistical analysis

Statistical parameters including the number of samples quantified, standard deviation, and statistical significance are described in figure legends. For two-group comparison, statistical significance was calculated by Student's *t*-test as described in figure legends. *, *p*<0.05; **, *p*<0.01; ***, *p*<0.001; ns, no significance.

## Results

### MORC2 is modified by SUMO1 and SUMO2/3

**Figure 1:**
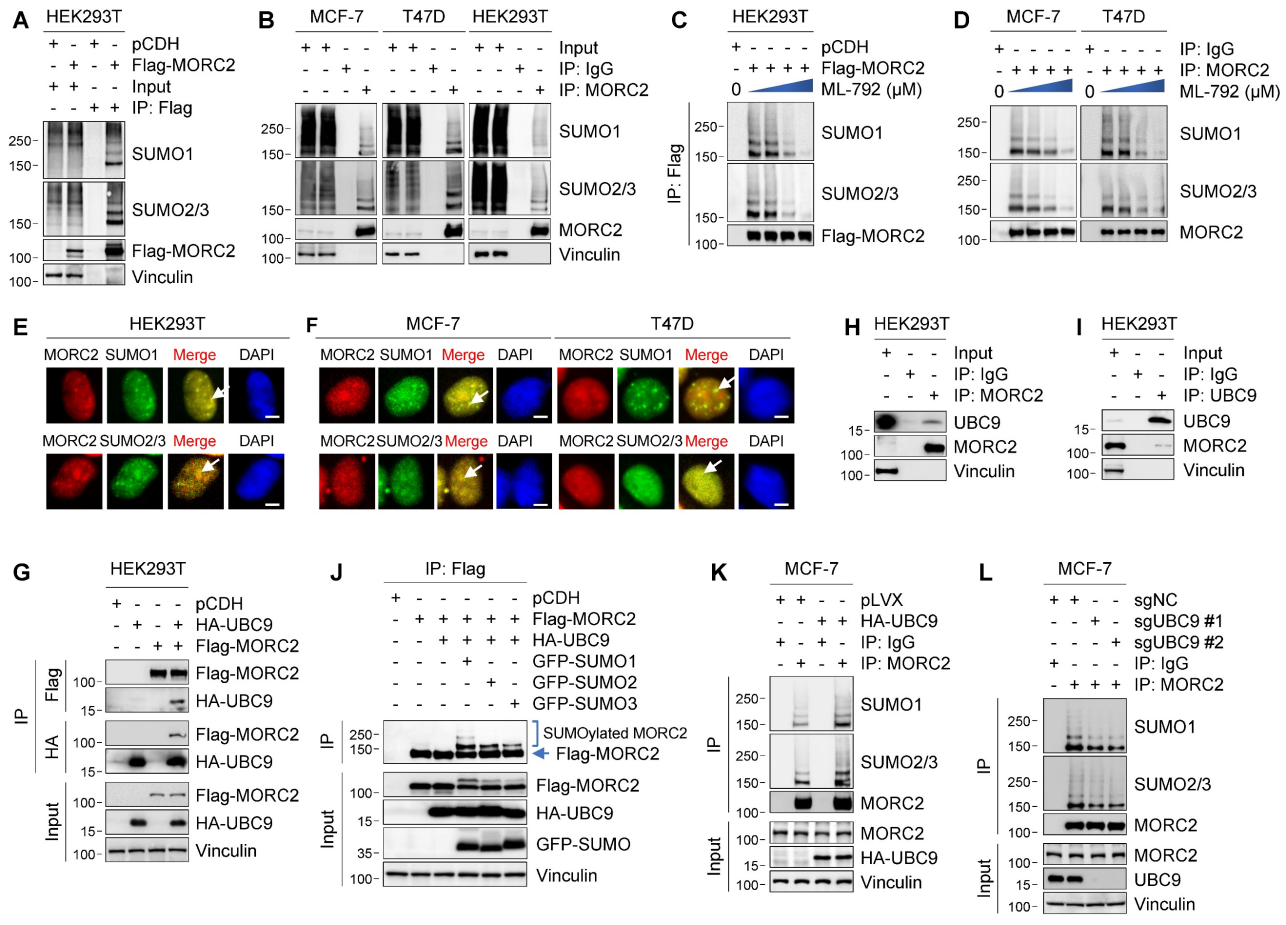
** MORC2 is modified by SUMO1 and SUMO2/3. (A)** Ectopically expressed Flag-MORC2 in HEK293T cells were pulled down with anti-Flag beads and analyzed by immunoblotting with indicated antibodies. **(B)** Endogenous MORC2 in MCF-7, T47D, and HEK293T cells were pulled down with an antibody against MORC2 and detected by immunoblotting with indicated antibodies. **(C)** Cells were treated with increasing doses of ML-792 (0, 0.01, 0.1 and 1 μM). Flag-MORC2 were pulled down with anti-Flag beads and analyzed by immunoblotting with antibodies against SUMO1 and SUMO2/3. **(D)** Endogenous MORC2 in MCF-7 and T47D cells was pulled down with an anti-MORC2 antibody and immunoblotted with indicated antibodies after treatment with increasing doses of ML-792 (0, 0.01, 0.1 and 1 μM). **(E-F)** HEK293T(E), MCF-7, and T47D cells (F) were fixed with paraformaldehyde solution and subjected to immunofluorescent staining with the indicated antibodies. Nuclei were counterstained with DAPI. Scale bar: 2.5 μm. **(H-I)** Total cellular lysates were subjected to IP assays with an anti-MORC2 (H) or anti-UBC9 (I) antibody, followed by immunoblotting using the indicated antibodies. **(G)** HEK293T cells were transfected with Flag-MORC2 and HA-UBC9 alone or in combination. Total cellular lysates were subjected to IP assays with anti-HA- or anti-Flag beads, followed by immunoblotting with the indicated antibodies. **(J)** HEK293T cells were transfected with Flag-MORC2, HA-UBC9 together with GFP-SUMO1, GFP-SUMO2 or GFP-SUMO3, respectively. Cellular lysates were pulled down with anti-Flag beads and then analyzed by immunoblotting. **(K)** MCF-7 cells were transiently transfected with or without HA-UBC9. Cellular lysates were used to IP assays with an anti-MORC2 antibody or control IgG, followed by immunoblotting with the indicated antibodies. **(L)** Endogenous UBC9 were knocked out in MCF-7 cells using two sgRNAs by the CRISPR/Cas9 system. Immunoprecipitated MORC2 were subjected to SUMOylation analysis.

Recently, several system-wide mass spectrometry analyses indicate that MORC2 is a potential SUMOylation target 18, 41, 42, but its regulatory mechanism and biological functions remain unexplored. To determine whether MORC2 is modified by SUMOylation, we transiently transfected Flag-MORC2 into HEK293T cells and conducted SUMOylation assays using anti-Flag beads under denature condition. Immunoblotting analysis with an anti-SUMO1 or anti-SUMO2/3 antibody revealed that SUMO1 and SUMO2/3 were conjugated to MORC2 (**Figure 1A**). Moreover, conjugation of SUMO1 and SUMO2/3 to endogenous MORC2 was detected in MCF-7, T47D, and HEK293T cells (**Figure 1B**). Consistently, incubation with SUMOylation inhibitor ML-792 20 resulted in a decrease in SUMOylation of ectopically expressed Flag-MORC2 in HEK293T cells (**Figure 1C** and **Figure S1A**) and of endogenous MORC2 in MCF-7 and T47D cells (**Figure 1D** and **Figure S1B**) in a dose-dependent manner. Immunofluorescent staining showed that endogenous SUMO1 and SUMO2/3 were co-localized with exogenously expressed Flag-MORC2 in HEK293T cells and with endogenous MORC2 in MCF-7 and T47D cells (**Figure 1E-F**, respectively; yellow color in the merged images indicated by white arrows). These results suggest that all of three SUMO isoforms are covalently conjugated to MORC2 in mammalian cells.

As UBC9 is the sole E2-conjugating enzyme essential for SUMOylation 10, we next investigated whether MORC2 interacts with UBC9 by reciprocal IP assays. As shown in **Figure 1G**, Flag-MORC2 was immunoprecipitated with HA-UBC9 when both were co-expressed in HEK293T cells. More importantly, an interaction between MORC2 and UBC9 at the endogenous protein level was detected in HEK293T cells (**Figure 1H**-**I)**. We also detected that all of three SUMO isoforms (GFP-tagged SUMO1, SUMO2, and SUMO3) were conjugated to MORC2 in the presence of HA-UBC9 **(Figure 1J)**. As expected, ectopic expression of HA-UBC9 enhanced, whereas knockout of endogenous UBC9 by CRISPR/Cas9 technology reduced, SUMOylation of endogenous MORC2 in MCF-7 cells (**Figure 1K**-**L**, respectively). Together, these results suggest that MORC2 is a SUMOylated protein.

### Lysine 767 (K767) is the major SUMOylation site in MORC2

To identify the potential MORC2 SUMOylation site(s), we used GPS (group-based prediction system)-SUMO 43 and JASSA (Joint Analyzer of SUMOylation site and SIMs) 44 programs to predict SUMOylation sites of MORC2. By overlapping the sites predicted by both algorithms, we narrowed down the potential SUMOylation sites in MORC2 to lysine 767 (K767) and K827 (**Figure S2A**-**B**). In addition, both sites were located within the canonical consensus SUMO motif Ψ-K-X-E, where Ψ is any hydrophobic amino acid and X is any amino acid 10 (**Figure S2A**).

**Figure 2:**
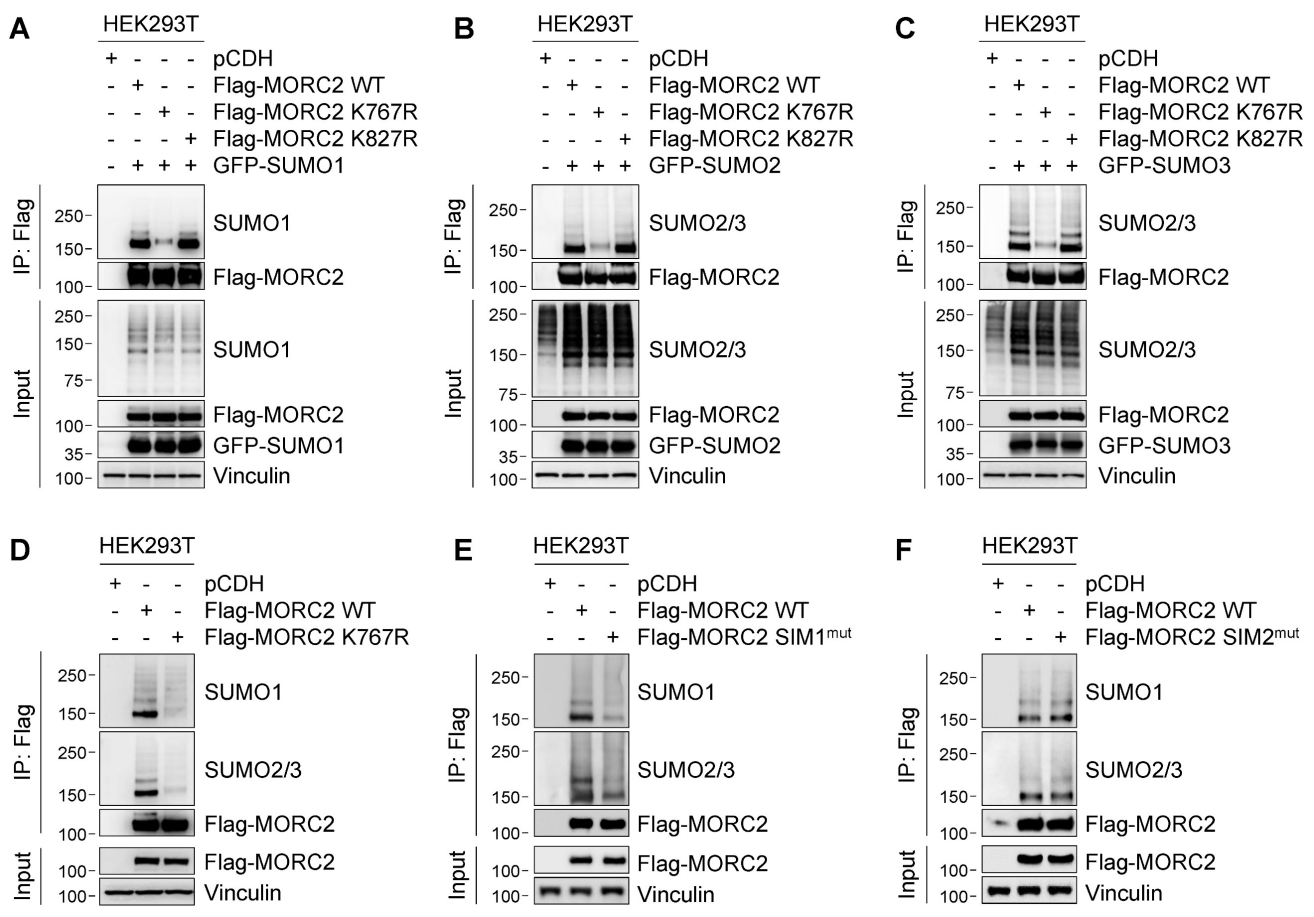
** K767 is the major SUMOylated site of MORC2. (A-C)** HEK293T cells were transfected with GFP-SUMO1 and Flag-MORC2 (WT, K767R, or K827R. Cells were lysed, and the ectopically expressed MORC2 was pulled down using anti- Flag, followed by immunoblotting with the indicated antibodies. Similar to A, SUMOylation levels of ectopically expressed MORC2 were analyzed in the presence of GFP-SUMO2 (B) or GFP-SUMO3 (C) overexpression, respectively. **(D)** Endogenous SUMO modification of ectopically expressed WT or K767R Flag-MORC2 was detected using the indicated antibodies. **(E-F)** The predicated SIM1 (residues 144-148) and SIM2 (residues 413-417) were mutated by substituting hydrophobic residues with alanine, termed SIM1^mut^ and SIM2^mut^, respectively. Then, SUMO modification of SIM1^mut^ and WT Flag-MORC2 (E) as well as SIM2^mut^ and WT Flag-MORC2 (F) was detected using the indicated antibodies.

To validate whether MORC2 is SUMOylated at those two sites, we individually mutated K767 and K827 to non-SUMOylable residue arginine (R), and then transiently transfected wild-type (WT), K767R, or K827R mutant Flag-MORC2 together with GFP-SUMO1, GFP-SUMO2, or GFP-SUMO3 into HEK293T cells. SUMOylation analyses with the indicated antibodies revealed that reduced SUMO1 and SUMO2/3 modification was observed in cells expressing K767R, but not K827R, mutant MORC2, as compared with its WT counterpart (**Figure 2A-C**, respectively). Moreover, conjugation of endogenous SUMO1 and SUMO2/3 to K767R mutant Flag-MORC2 was decreased compared to WT MORC2 (**Figure 2D**). These results suggest that K767 is the main SUMO site of MORC2. Sequence alignment revealed that the K767 residue was highly conserved across various species (**Figure S2C**). Immunofluorescent staining showed that both WT and K767R mutant MORC2 were localized mainly in the nucleus (**Figure S2D**). Together, these results suggest that MORC2 SUMOylation mainly occurs at K767 and does not affect its subcellular localization.

### A functional SIM at the N-terminus of MORC2 is required for its efficient SUMOylation

It has been documented that the SIM is required for efficient SUMOylation of some SUMO substrates 15, 16. Analysis of MORC2 protein sequence using GPS-SUMO 43 and JASSA 44 programs revealed the presence of two putative SIMs in MORC2, termed SIM1 and SIM2, which are located within the residues 144-148 and 413-417, respectively (**Figure S2E**). To assess the functional importance of those two SIMs in MORC2 SUMOylation, we individually mutated two SIM sequences by substitution of hydrophobic residue with alanine 15 (herein referred to as SIM1^mut^ and SIM2^mut^). SUMOylation assays revealed that Flag-MORC2 SIM1^mut^, but not SIM2^mut^, had reduced MORC2 SUMOylation (**Figure 2E**-**F**, respectively). Moreover, the SIM1 sequence was highly conserved across multiple species (**Figure S2C**). Mutation of the SIM1 sequence did not affect subcellular localization of MORC2 (**Figure S2F**). Collectively, these data indicates that the SIM1 in MORC2 is functional and contributes to its efficient SUMOylation.

### TRIM28 functions as the SUMO E3 ligase for MORC2 SUMOylation

SUMO E3 ligases are critical for determining the specificity and function of SUMOylation substrates 10. Analysis of protein interaction database BioGRID (https://thebiogrid.org) 45 found that two putative SUMO E3 ligases, tripartite motif containing 28 (TRIM28; also known as KAP1 or TIF1B) 46 and chromobox 4 (CBX4) 47, are potential binding partners of human MORC2 (**Figure S3**, indicated by red circles). Examination of our recent proteomic results of MORC2 interactome 28 also found that TRIM28 is a potential interactor of MORC2 (**Figure S4A**). Although Flag-MORC2 was associated with endogenous CBX4 in HEK293T cells (**Figure S4B**), ectopic expression of Flag-CBX4 did not significantly affect Flag-MORC2 SUMOylation (**Figure S4C**), indicating that CBX4 is not a SUMO E3 ligase for MORC2 SUMOylation.

**Figure 3:**
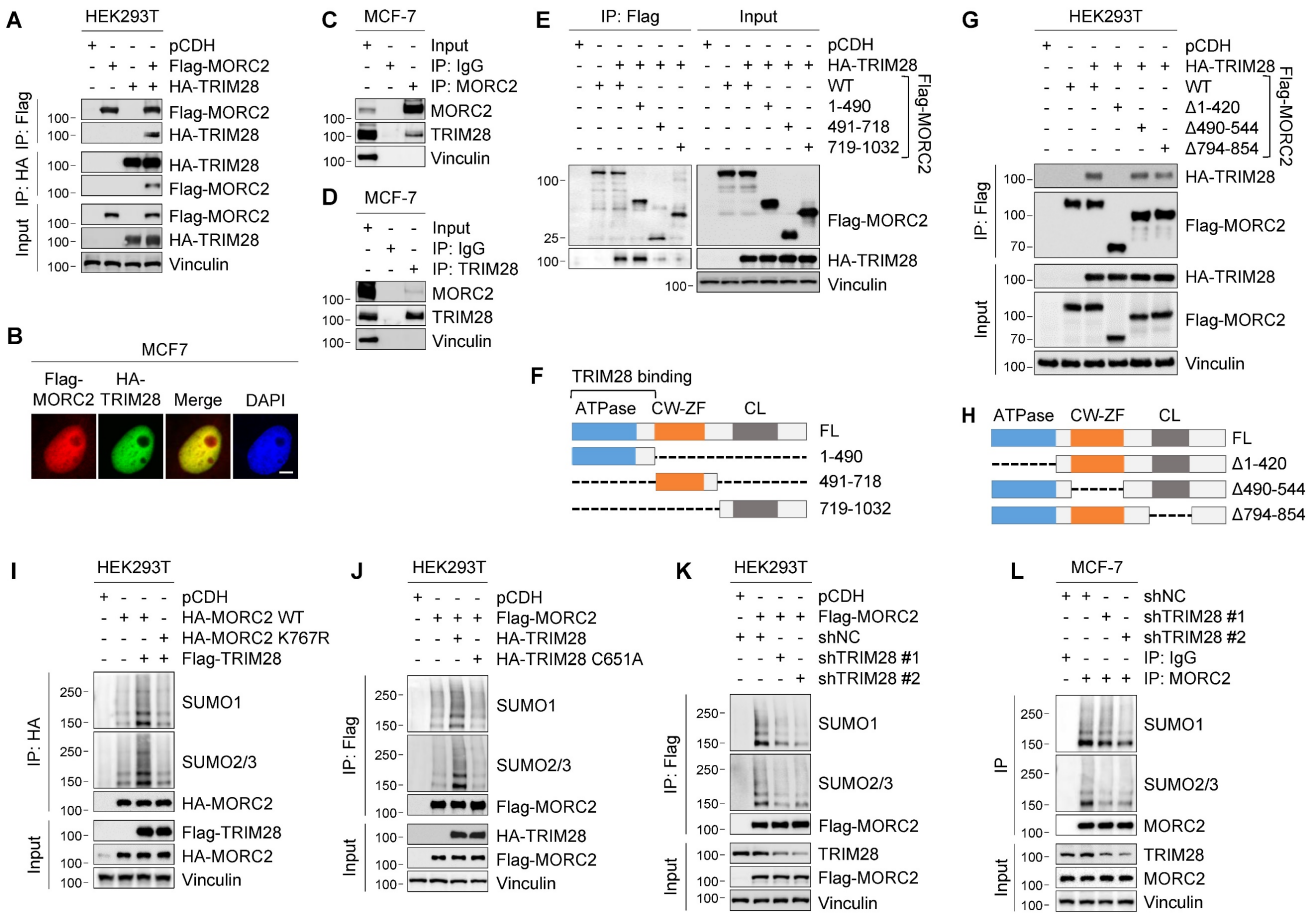
** TRIM28 acts as SUMO E3 ligase for MORC2. (A)** HEK293T cells were transfected with Flag-MORC2 and HA-TRIM28 alone or in combination. Lysates were used to IP assays with anti-Flag- or anti-HA beads, followed by immunoblotting analysis with the indicated antibodies. **(B)** Co-localization of MORC2 and TRIM28 was analyzed by immunofluorescent staining with the indicated antibodies. Nuclei were counterstained with DAPI. Scale bar: 2.5 μm. **(C-D)** Lysates of MCF-7 cells were subjected to IP assays with control IgG, anti-MORC2 (C) or anti-TRIM28 (D) antibody, followed by immunoblotting analysis. **(E-F)** HEK293T cells were transfected with the indicated expression vectors. After 48 h of transfection, IP and immunoblotting analyses were carried out with the indicated antibodies (E). Schematic diagram showing the region of MORC2 for TRIM28 binding (F). **(G-H)** HEK293T cells were transfected with the indicated expression vectors. After 48 h of transfection, IP and immunoblotting analyses were carried out with the indicated antibodies (G). Schematic diagram showing the deletion region of MORC2 (H). **(I)** HEK293T cells were transfected with WT or K767R HA-MORC2 alone or in combination with Flag-TRIM28. Cellular lysates were pulled down with anti-HA beads, and then subjected to detect the SUMOylation levels of HA-MORC2. **(J)** HEK293T cells were transfected with Flag-MORC2 alone or in combination with WT or C651A mutant HA-TRIM28. IP assays were performed with anti-Flag beads, followed by immunoblotting analysis to detect the SUMOylation levels of MORC2. **(K)** HEK293T cells were transfected with Flag-MORC2 along with nonspecific control shRNA (shNC) or two different shRNAs targeting TRIM28. IP assays were performed with anti-Flag beads, followed by immunoblotting analysis with the indicated antibodies. **(L)** MCF-7 cells were transfected with shNC or two different shRNAs targeting TRIM28. IP assays were carried out with an anti-MORC2 antibody, followed by immunoblotting analysis with the indicated antibodies.

We next examined whether TRIM28 induces MORC2 SUMOylation. IP and immunoblotting assays showed that Flag-MORC2 was co-immunoprecipitated with HA-TRIM28 in HEK293T cells (**Figure 3A**). Co-localization of Flag-MORC2 and HA-TRIM28 was also observed in the nuclei of MCF-7 cells revealed by immunofluorescent staining (**Figure 3B**). Moreover, an association between endogenous MORC2 and endogenous TRIM28 was detected in MCF-7 cells (**Figure 3C**-**D**). GST pull-down assays showed that MORC2 and TRIM28 can interact directly (**Figure S5A**).

To map which domain of MORC2 is required for its interaction with TRIM28, we generated three MORC2 deletion and truncation constructs and performed co-IP assays in HEK293T cells. As shown in **Figure 3E**-**F**, The N-terminus of MORC2 containing the conserved ATPase domain (residues 1-490) was required for its interaction with TRIM28. Consistently, MORC2 with deletion of residues 1-420 at its N-terminus was unable to bind to TRIM28 (**Figure 3G-H**). These results suggest that the N-terminal ATPase domain (residues 1-420) of MORC2 is essential for its interaction with TRIM28. It has been reported that the SIM motif can enhance SUMOylation by promoting the E3-SUMO-SIM interaction. Interestingly, we noticed the SIM1 motif (residues 144-148) is localized within the MORC2 ATPase domain (**Figure S2B**), indicating the importance of SIM1 motif in the function of MORC2. Collectively, these results suggest that TRIM28 physically interacts with MORC2.

To address whether TRIM28 is required for MORC2 SUMOylation, we ectopically expressed HA-TRIM28 and detected MORC2 SUMOylation levels. As shown in **Figure 3I**, ectopic TRIM28 markedly enhanced SUMOylation of WT but not K767R mutant MORC2. In addition, over-expression of TRIM28 did not significantly affect MORC2 protein levels (**Figure 3I**). As a control, we also examined the effects of 5 members of the PIAS (protein inhibitor of activated STAT) family of SUMO E3 ligases 48 on MORC2 SUMOylation. As shown in **Figure S4D,** HA-TRIM28 had more pronounced effects on MORC2 SUMOylation than HA-PIAS1, 2a, 2b, 3, and 4 did (compare lane 8 and others). Moreover, only overexpression of WT TRIM28, but not its catalytically inactive C651A mutant 49, effectively enhanced MORC2 SUMOylation (**Figure 3J**, compare lane 3 and 4). Conversely, knockdown of TRIM28 by two independent shRNAs (shTRIM28 #1 and #2) dramatically reduced SUMOylation of ectopically expressed Flag-MORC2 in HEK293T cells (**Figure 3K**) and of endogenous MORC2 in MCF-7 cells (**Figure 3L**). In addition, *in vitro* SUMOylation assays also indicated that TRIM28 enhanced MORC2 SUMOylation (**Figure S5B**). Collectively, these results suggest that TRIM28 is the SUMO E3 ligase required for MORC2 SUMOylation.

### SENP1 is a MORC2 deSUMOylase

**Figure 4:**
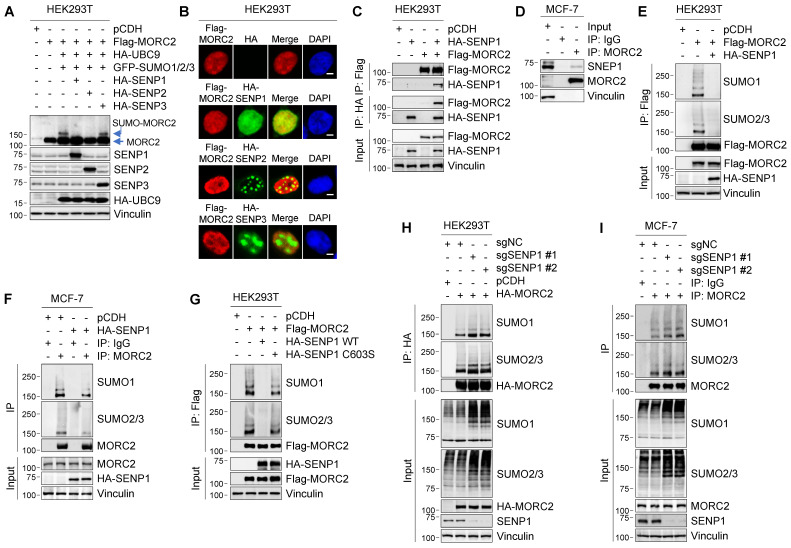
**SENP1 serves as a deSUMOylase for MORC2. (A)** HEK293T cells were transfected with the indicated expression vectors. Cellular lysates were analyzed by immunoblotting. **(B)** Co-localization of MORC2 with HA-SENP1, HA-SENP2 or HA-SENP3 was analyzed by immunofluorescent staining with the indicated antibodies. Nuclei were counterstained with DAPI. Scale bar: 2.5μm. **(C)** HEK293T cells were transfected with Flag-MORC2 and HA-SENP1 alone or in combination. Cellular lysates were subjected to IP assays with anti-Flag or anti-HA beads, followed by immunoblotting analysis with the indicated antibodies. **(D)** Lysates from MCF-7 cells were processed for IP assays using an anti-MORC2 antibody, and immunoblotted with indicated antibodies. **(E-F)** HEK293T (E) and MCF-7 (F) cells were transfected with the indicated expression vectors. The sequential IP and immunoblotting analyses were conducted with the indicated antibodies. **(G)** HEK293T cells were transfected with Flag-MORC2 alone or in combination with WT or C603S HA-SENP1. The sequential IP and immunoblotting analyses were conducted with the indicated antibodies. **(H-I)** Endogenous SENP1 was knocked out using the CRISPR/Cas9 system in HEK293T cells expressing HA-MORC2 (H) or in MCF-7 cells (I). The sequential IP and immunoblotting analyses were conducted with the indicated antibodies.

Protein SUMOylation is reversed by 6 SENPs in mammalian cells, including SENP1-3 and SENP5-7 10. Of note, SENP1 and SENP2 can deconjugate all SUMO isoforms, whereas SENP3 and SENP5-7 preferentially deconjugate SUMO2/3-modified proteins and SUMO chains 10. As MORC2 is modified by all three SUMO isoforms (**Figure 1**), we next examined the potential role of SENP1-3 in MORC2 deSUMOylation. To do this, Flag-MORC2, HA-UBC9, and GFP-SUMOs were transfected into HEK293T cells in the presence or absence of HA-SENP1, 2, or 3. Results showed that SUMOylation of MORC2 was attenuated by co-transfection with SENP1 and SENP2 but not SENP3 (**Figure 4A**). Immunofluorescent staining showed that HA-SENP1 was co-localized with Flag-MORC2 in the nucleus, whereas HA-SENP2 and HA-SENP3 were not (**Figure 4B**). In addition, MORC2 did not interact with SENP2 (**Figure S6A**-**B**). Based on these data, we next focused on addressing the role of SENP1 in MORC2 deSUMOylation.

To determine whether SENP1 mediates MORC2 deSUMOylation, we examined the interaction between SENP1 and MORC2. IP assays demonstrated that SENP1 interacted with MORC2 (**Figure 4C**). The interaction between SENP1 and MORC2 at the endogenous protein level was observed in MCF-7 cells (**Figure 4D**). It was also found that the MORC2 ATPase domain was required for this interaction (**Figure S6C**). In addition, SENP1 overexpression dramatically decreased SUMOylation of ectopically expressed MORC2 in HEK293T cells and endogenous MORC2 in MCF-7 cells (**Figure 4E**-**F**). The enhanced MORC2 SUMOylation, induced by TRIM28 or SUMO overexpression, was reversed by co-expression of SENP1 (**Figure S6D**-**E**). More importantly, ectopic expression of WT SENP1, but not its catalytically inactive C603S mutant 50, effectively abolished MORC2 SUMOylation (**Figure 4G**). Consistently, depletion of SENP1 remarkably induced MORC2 SUMOylation in both HEK293T (**Figure 4H**) and MCF-7 cells (**Figure 4I**). In addition, *in vitro* SUMOylation assays also showed that SENP1 significantly blocked SUMOylation of MORC2 (**Figure S5C**). Taken together, these results indicate that SENP1 is the primary SENP for MORC2 deSUMOylation.

### SUMOylation of MORC2 is decreased following DNA damage

As SUMOylation has been implicated in DDR 51, we next measured whether DNA-damaging agents influence MORC2 SUMOylation. Toward this aim, we treated HEK293T cells stably expressing pCDH and Flag-MORC2 with or without the following DNA-damaging agents, including adriamycin (ADR), cisplatin (CDDP), etoposide (VP-16), methyl methane sulfonate (MMS), and hydrogen peroxide (H_2_O_2_), for 2 h, and then assessed their effects on MORC2 SUMOylation. As shown in **Figure S7A**, these DNA-damaging agents effectively induced DNA damage as evidenced by a significant upregulation of DNA damage marker γH2AX (phosphorylation of H2AX at Ser 139) 52, and simultaneously decreased MORC2 SUMOylation. Treatment of HEK293T cells with ADR for 2 h resulted in a decrease in MORC2 SUMOylation in a dose-dependent manner (**Figure S7B**).

Our previously studies have shown that MORC2 can be modified by phosphorylation at serine 739 (S739) 23 and by acetylation at K767 (K767Ac) 30 in response to DNA damage. We next examined whether there is a crosstalk between these PTMs. Results showed that the levels of MORC2 K767Ac were upregulated, whereas its SUMOylation levels were downregulated after treatment with ADR for 2 h (**Figure S7C**), indicating a negative crosstalk between MORC2 SUMOylation and acetylation at K767. Additionally, we also found that S739 phosphorylation of MORC2 was required for SUMOylation of MORC2 (**Figure S7D**). These data suggest that SUMOylation of MORC2 cooperates with other PTMs to regulate DDR.

**Figure 5:**
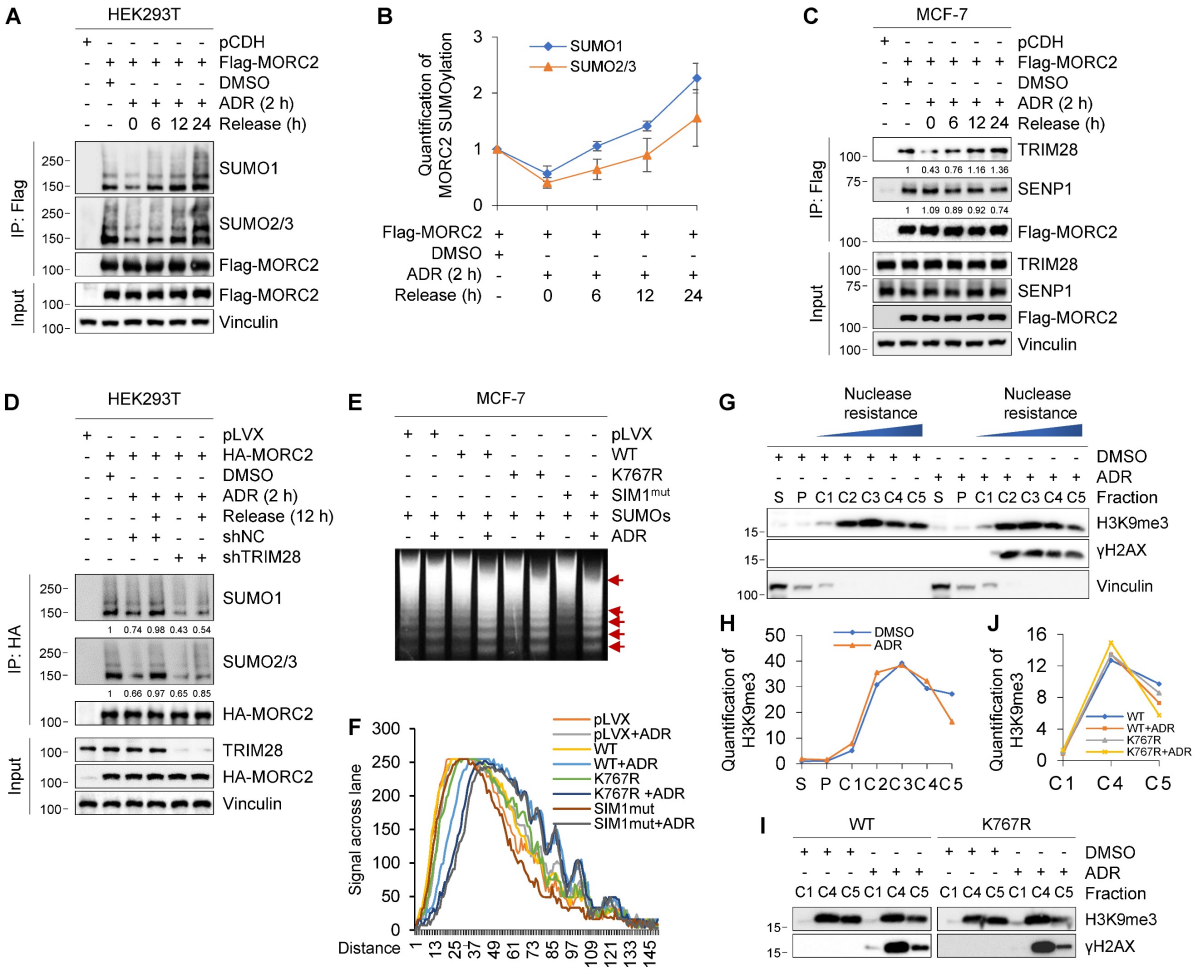
**Transient MORC2 deSUMOylation in response to genotoxic stress. (A-B)** HEK293T cells were transfected with Flag-MORC2 for 48 h and then treated with or without 1 μM ADR for 2 h, followed by release for the indicated times. Cellular lysates were subjected to IP assays using anti-Flag beads, followed by immunoblotting to detect the SUMOylation levels of MORC2 (A). Quantitative results of SUMO modification levels of MORC2 are shown in B. **(C)** MCF-7 cells stably expressing pCDH and Flag-MORC2 were treated with or without 1 μM ADR for 2 h, followed by release for the indicated times. Cellular lysates were subjected to IP assays with anti-Flag beads, followed by immunoblotting analysis. **(D)** HEK293T cells transfected with HA-MORC2 alone or in combination with shNC or shTRIM28 were treated with 1 μM ADR for 2 h, followed by release for 12 h. Lysates were subjected to IP assays with anti-HA beads, followed by immunoblotting analysis. **(E)** MCF-7 cells stably expressing control vector, WT, K767R or SIM1^mut^ MORC2 were treated with or without 1 μM ADR for 2 h, followed by MNase assays. The arrows indicate the positions of nucleosomal DNA ladders, including mono-, di-, tri- and multiple-nucleosomal DNA. **(F)** The quantification of signal in each lane of the gel was conducted using software ImageJ. **(G-H)** MCF-7 cells were treated with or without 1 μM ADR for 2 h, followed by fractionating into extracts as described in Materials and Methods. Immunoblotting was conducted using the indicated antibodies(G). Quantitative results of the indicated signal are shown in H. S, cytoplasmic fraction; P, nucleoplasmic fraction; C, nuclease-resistant fraction of nucleosomes. **(I-J)** MCF-7 cells stably expressing WT or K767R MORC2 were treated with 1 μM ADR for 2 h, and fractionated into extracts as described in G. Immunoblotting was conducted using the indicated antibodies (I). Quantitative results of H3K9me3 signal are shown in J.

Interestingly, we found that rapid loss of MORC2 SUMOylation caused by ADR was restored in HEK293T cells after recovery for the times indicated in **Figure 5A**-**B**. These results suggest that MORC2 SUMOylation is highly dynamic in response to DNA damage. To address underlying mechanism for the dynamic change of MORC2 SUMOylation in response to DNA damage, we next tested whether ADR affects the interaction of MORC2 with TRIM28 or SENP1. As shown in **Figure 5C**, treatment with ADR for 2 h did not significantly change the protein levels of TRIM28 or SENP1, but significantly reduced the interaction between MORC2 and TRIM28 but not SENP1. Interestingly, the impaired interaction between MORC2 and TRIM28 was restored after recovery for the indicated times (**Figure 5C**, compare lanes 4-6 with 3), accompanied by a slight decrease in the association of MORC2 with SENP1. Critically, knockdown of TRIM28 dramatically impaired MORC2 SUMOylation recovery after ADR treatment (**Figure 5D**). These data suggest that precisely regulated MORC2 SUMOylation depends mainly on TRIM28 in response to DNA damage.

Previous studies have shown that MORC2 exerts ATPase-dependent chromatin remodeling activity in response to DNA damage 23. We next determined whether MORC2 deSUMOylation during the early stage of DDR is related to its chromatin remodeling activity. To do this, we assessed the chromatin condensation by micrococcal nuclease (MNase) sensitivity assays, a method to detect chromatin accessibility 3. Damaged chromatin is relatively more accessible for efficient DDR and increases MNase susceptibility 53. Treatment of cells expressing WT MORC2 with ADR enhanced chromatin accessibility to MNase. Moreover, this effect was enhanced in cells expressing SUMOylation-defective MORC2 (K767R or SIM1^mut^) (**Figure 5E**-**F**). In addition, we applied chromatin segregation assays to identify the differential properties of chromatin. Nuclease-resistant chromatin fractions enrich histone 3 lysine 9 trimethylation (H3K9me3), a hallmark of heterochromatin formation, indicating highly condensed chromatic nucleosomes 40. As shown in **Figure 5G**-**H,** ADR treatment mobilized H3K9me3-rich nucleosomes from part C5 to the more nuclease-sensitive part C4. This discovery corroborated the widely held view that damaged chromatin is relatively more accessible. We then tested whether MORC2 SUMOylation participates in this process. Our findings were consistent with the results shown in **Figure 5G**. Expression of WT MORC2 decreased H3K9me3-enriched nucleosome chromatin in fraction C5 in response to ADR treatment. Furthermore, SUMOylation-defective MORC2 mutant enhanced this effect (**Figure 5I**-**J**). Therefore, the increase in chromatin accessibility dependent on MORC2 chromatin remodeling activity was enhanced by deSUMOylation following genotoxic damage, thus further promoting DNA repair.

### MORC2 SUMOylation contributes to DNA repair

**Figure 6:**
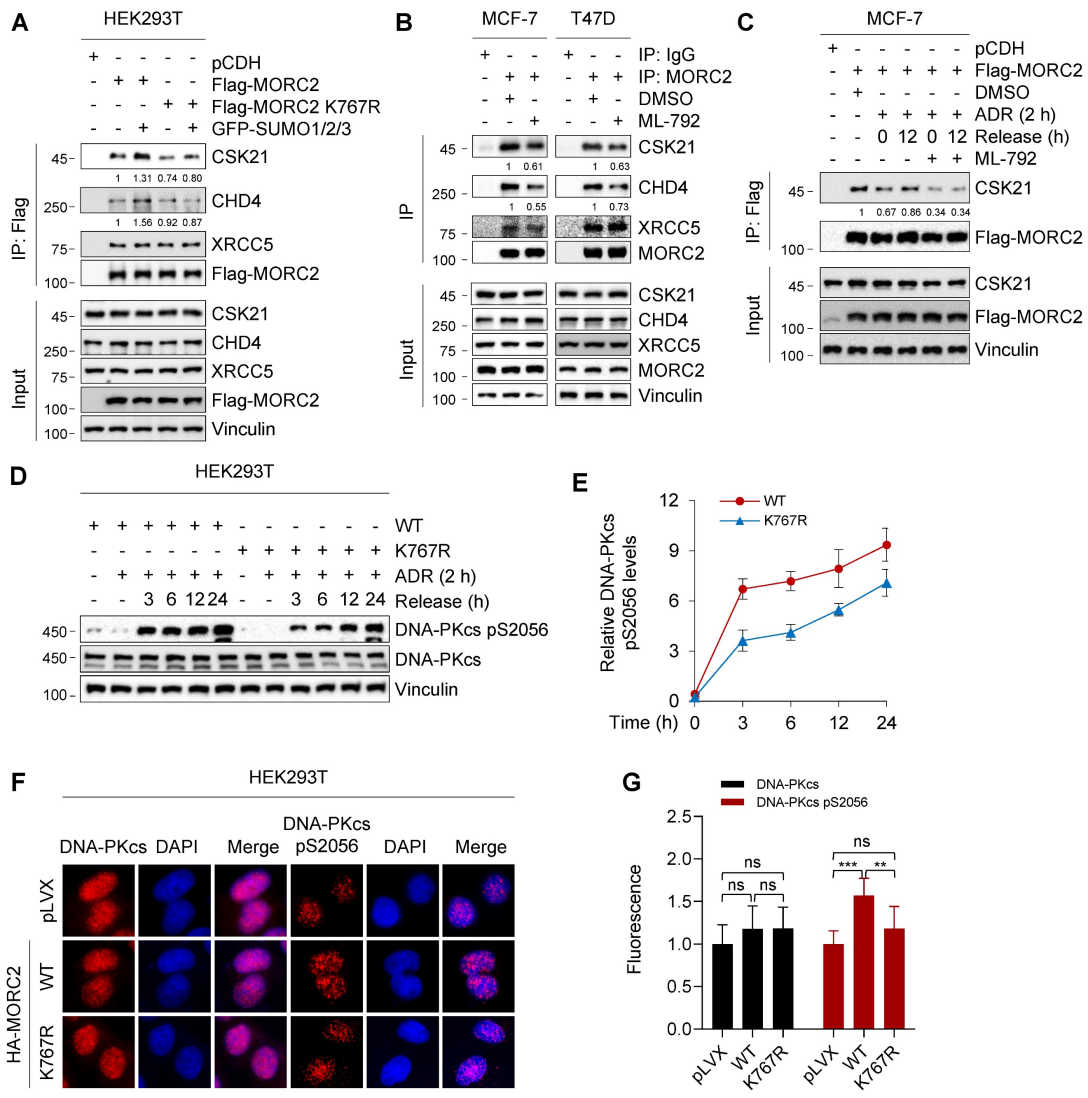
** SUMOylation of MORC2 contributes to DNA repair. (A)** HEK293T cells were transfected with WT or K767R MORC2 alone or in combination with GFP-SUMO plasmids for 48 h. Cellular lysates were subjected to IP assays using anti-Flag beads, followed by immunoblotting analysis. **(B)** MCF-7 and T47D cells were treated with or without 1 μM ML-792 for 24 h. Cellular lysates were subjected to IP assays using anti-MORC2 antibody, followed by immunoblotting analysis. **(C)** MCF-7 cells stably expressing Flag-MORC2 were treated with 1 μM ADR for 2 h, followed by release for 0 or 12 h, in the presence or absence of 1 μM ML-792. Cellular lysates were harvested for IP assays with anti-Flag beads, followed by immunoblotting analysis. **(D-E)** HEK293T cells stably expressing WT or K767R MORC2 were treated with 1 μM ADR for 2 h, followed by release for the indicated times, and then subjected to immunoblotting analysis (D). Relative expression levels of DNA-PKcs p2056 (DNA-PKcs pS2056/Vinculin) are shown in E. **(F-G)** HEK293T cells stably expressing control vector, WT or K767R MORC2 were treated with 1 μM ADR for 2 h, followed by recovery for 24 h, and subjected to immunofluorescent staining with the indicated antibodies (F). Quantification analysis was conducted using ImageJ software, followed by statistical analysis with Student's *t* test (G).

An intact SUMOylation cycle of MORC2 is important for cell survival in response to genotoxic stress. It was reported that conjugated SUMO molecules exert a wide range of effects on substrates, alter substrate interactions and modulate their functions in various biological processes 54, 55. We then investigated whether MORC2 SUMOylation changes its interactions and functionally contributes to DNA repair. We used co-IP assays coupled with LC-MS/MS to identify the SUMOylated MORC2 interactome (**Figure S8A**). To optimize specificity, proteins only detectable in co-IP samples from HEK293T cells transfected with WT-MORC2 and GFP-SUMOs were classified as SUMOylated MORC2-interacting proteins (**Figure S8B**). Among the binding proteins, the functions of protein kinase CSK21 (casein kinase II subunit alpha), CHD4 (chromodomain helicase DNA binding protein 4), and XRCC5 (X-ray repair cross complementing 5) are closely associated with DNA repair (**Figure S8C**). To validate these results, we conducted IP analyses and found that MORC2 SUMOylation significantly increased interactions of MORC2 with CSK21 and CHD4, whereas SUMOylation-defective MORC2 mutants greatly impaired these interactions even in the presence of SUMO overexpression (**Figure 6A**). Treatment of MCF-7 and T47D cells with SUMOylation inhibitor ML-792 20 decreased MORC2 SUMOylation, which in turn significantly decreased the interaction of MORC2 with CSK21 and CHD4 (**Figure 6B**). These findings prompted us to test whether the restoration of MORC2 SUMOylation after ADR treatment could increase the affinities for SUMO-MORC2 binding with CSK21 and CHD4, thereby enhancing DNA repair.

CSK21 is a clinically targetable serine/threonine kinase that participates in drug resistance and DNA repair 56. We first tested whether CSK21 is the MORC2 SUMOylation binding effector. Attenuation of the interaction between MORC2 and CSK21 following ADR treatment was recovered after ADR release (**Figure 6C**; compare lane 3 with 4), but ML-792 treatment significantly impaired binding recovery (**Figure 6C**; compare lane 5 with 6). In addition, ubiquitination of MORC2 did not affect its interaction with CSK21(**Figure S9A**). Therefore, deficiency of MORC2 SUMOylation diminished CSK21 recruitment and might not favor downstream factor further activation. DNA-dependent protein kinase catalytic subunit (DNA-PKcs) is an important kinase that responds to DNA damage, and is activated by CSK21 to promote DNA repair 57. We observed DNA-PKcs activation in WT and K767R mutant MORC2 by treating the cells with ADR for 2 h and releasing them for the indicated times. **Figure 6D**-**E** show that, compared to K767R mutant, WT MORC2 markedly increased DNA-PKcs phosphorylation at serine 2056 without affecting its total protein levels. This result was confirmed by immunofluorescent staining (**Figure 6F**-**G**). Furthermore, we found that PPARG, also known as peroxisome-proliferator receptor-γ (PPARγ), which modulates cellular response to DNA damage and make cancer cells resistant to cytotoxic chemotherapy 58, acted as the downstream target of MORC2 SUMOylation to participate in the DNA damage repair process (**Figure S9B-C**). The foregoing data suggest that MORC2 SUMOylation may promote efficient DNA repair partially through CSK21-induced DNA-PKcs activation.

### MORC2 SUMOylation is required for cell survival in response to DNA-damaging agents

**Figure 7:**
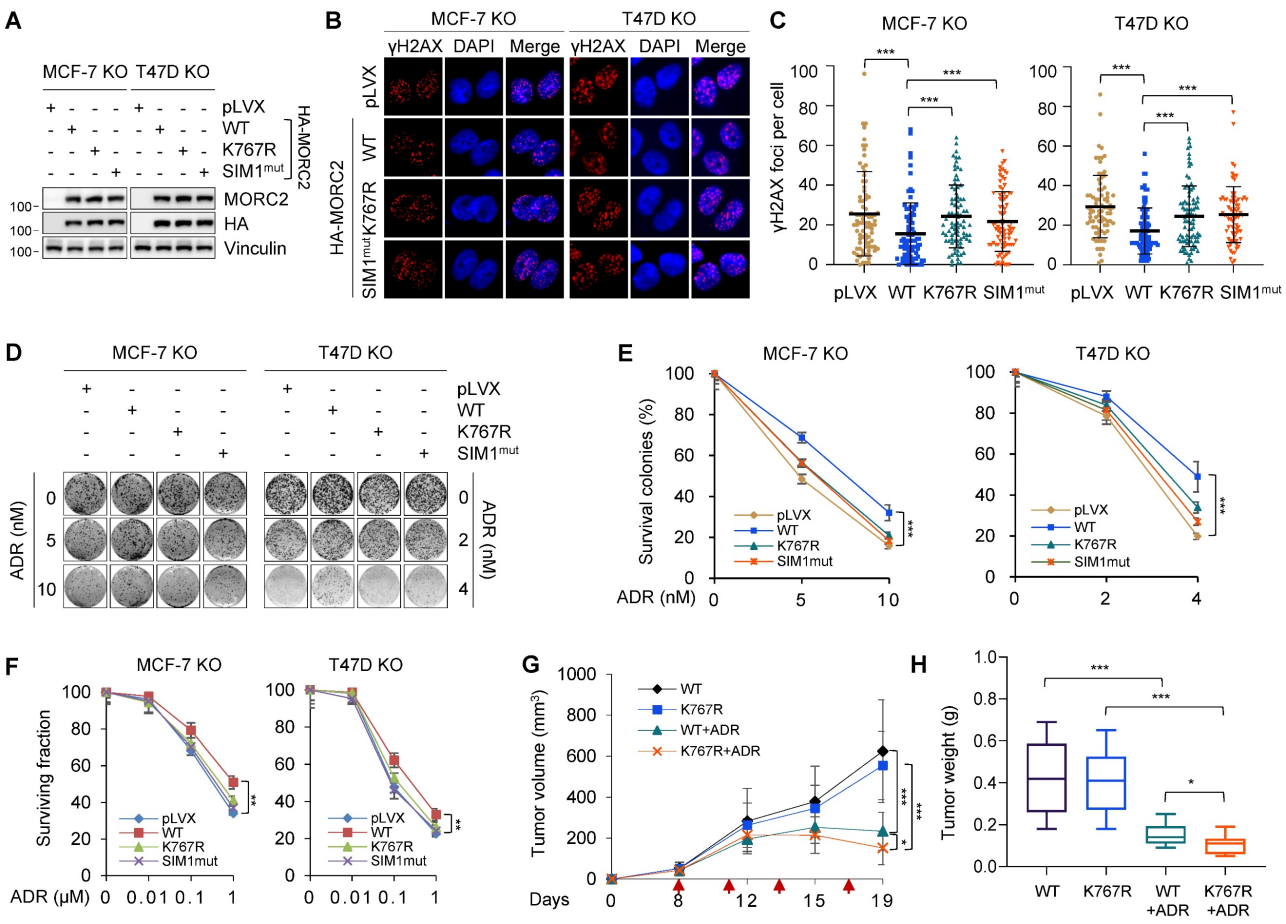
** MORC2 SUMOylation facilitates cell survival against genotoxic stress. (A)** MORC2-knockout MCF-7 and T47D cells were reconstituted with WT, K767R and SIM1^mut^ MORC2 through lentiviral infection. Comparable levels of reconstituted MORC2 were validated by immunoblotting. **(B-C)** The established MCF-7 and T47D cells (A) were treated with 1 μM ADR for 0.5 h and released for 24 h before immunofluorescent staining for γH2AX or DAPI. Representative images (B) and corresponding quantitative results (C) are shown. **(D-E)** The established MCF-7 and T47D cells (A) were treated with or without the indicated doses of ADR for 14 days. The representative images of survival colonies are shown in D, and quantitative results (E) are represented as mean ± S.D. as indicated (n=3). **(F)** The established MCF-7 and T47D cells (A) were treated with the increasing doses of ADR as indicated for 72 h, and then subjected to CCK-8 assays. Quantitative results are represented as mean ± S.D. as indicated (n=3). **(G)** MORC2-knockout LM2-4175 cells were reconstituted with WT or K767R MORC2. The established LM2-4175 cells were injected into the subcutaneous flanks of female nude mice to establish xenograft tumors (n=14/group). Chemotherapy ADR treatment was started when the tumor volume in one of the groups exceeded 100 mm^3^. Mice were treated with 3 mg/kg ADR intraperitoneally on days as red arrows highlighted. Tumor volume and growth kinetics were measured over time. **(H)** Tumors were harvested and weighed after 19 days of ADR treatment. Statistical significance was assessed by Student's *t*-test.

We next investigated whether SUMOylation mediates the biological function of MORC2 in the DNA repair process and thus affects cell survival in response to genotoxic stress. To eliminate the potential effects of endogenous MORC2, we knocked out endogenous MORC2 in MCF-7 and T47D cells and then reconstituted WT, SUMOylation-deficient mutant K767R, and SIM1^mut^ MORC2 by lentiviral infection (**Figure 7A**). Cells were treated with DNA-damaging agent ADR for 30 min and then recovered for 24 h. The levels of phosphorylated histone H2AX on Ser139 (γH2AX) were examined by immunofluorescent staining. Prolonged presence of γH2AX post DNA damage indicates defective DNA repair. As shown in **Figure 7B-C**, cells expressing K767R and SIM1^mut^ mutant MORC2 had higher levels of γH2AX foci after 24 h of recovery than cells expressing WT MORC2 did, suggesting that SUMOylation-deficient mutants induce inefficiency in clearing DNA lesions. Hence, MORC2 SUMO modification is necessary for efficient DNA repair. Colony formation assays showed that WT MORC2, but not K767R or SIM1^mut^ mutants, reduced cellular sensitivity to ADR (**Figure 7D**-**E**) and another DNA-damaging agent MMS (**Figure S10A-B**). Consistently, CCK-8 survival assays indicated that SUMOylation of MORC2 displayed similar effects on cellular sensitivity to ADR (**Figure 7F**). These data suggests that MORC2 SUMOylation is required for cell survival in response to genotoxic stress.

To test whether MORC2 SUMOylation is important in resistance of breast cancer cells to ADR, we established xenograft tumor models with human LM2-4175 cells 59. MORC2-depleted LM2-4175 cells with stably reconstituted WT or K767R MORC2 were subcutaneously injected into nude mice (**Figure S11A**). Mice were then intraperitoneally administered 3 mg/kg ADR and monitored for xenograft tumor growth. As shown in **Figure 7G-H** and **S11B**, tumors expressing K767R mutant MORC2 were significantly smaller and lighter than those expressing WT MORC2 after ADR treatment, indicating that that MORC2 K767R mutant cells were more sensitive to ADR. Immunohistochemistry (IHC) staining of apoptosis maker cleaved caspase 3 revealed that tumors expressing K767R mutant MORC2 displayed increased cleaved caspase 3 as compared with those expressing WT MORC2 (**Figure S11C**-**D**). This finding prompted us to test whether treatment of cells with ML-792 would enhance the sensitivity of breast cancer cells to ADR treatment. As shown in **Figure S11E,** ML-792 markedly impaired WT MORC2-indcued ADR resistance. Therefore, SUMO modification-deficient MORC2 results in more severe DNA damage and renders breast tumors more sensitive to ADR than WT MORC2. For this reason, MORC2 SUMOylation is critical in breast tumor resistance to DNA-damaging chemotherapy.

## Discussion

SUMOylation plays important roles in cancer as it is vital to DDR and the maintenance of genome integrity 51. Clarifying the mechanism of this fine-tuned post-translational modification process and identifying the functional targets can help hasten the development of novel anticancer therapeutic strategies. Accumulating evidence shows that dysregulation of SUMOylation has been linked with several human diseases, including neurodevelopmental disability 60 and cancer progression 32. However, its regulatory mechanisms remain largely unknown. In the present study, we found that dynamic SUMOylation of MORC2 by TRIM28 orchestrates chromatin remodeling and DNA repair in response to DNA damage and drives chemoresistance in breast cancer.

First, we report for the first time that MORC2 is modified by SUMO1 and SUMO2/3 at K767 in a SIM-dependent manner, and this process is control by SUMO E3 ligase TRIM28 and deSUMOylase SENP1 (**Figures 1-4**). Previous studies have shown that some proteins are modified only by SUMO1 or SUMO2/3, but others are modified by all of three SUMO paralogs 13. For instance, DBC1 is specifically modified by SUMO2/3 61, whereas dual-specificity phosphatase 6 (DUSP6) is modified by SUMO1 and SUMO2/3 62. TRIM28 as a SUMO E3 ligase has been shown to modify substrate proteins, such as IFN regulatory factor 7 (IRF7) 46, NPM1/B23 63, CDK9 64, and proliferating cell nuclear antigen (PCNA) 65. DeSUMOylation is catalyzed by the members of the SENP family. In this context, SENP1 and SENP2 cleave all three SUMO isoforms from substrates, while SENP3 and SENP5 are dedicated to detach mainly SUMO2/3 from its substrates 66. We found that SENP1 is a MORC2 deSUMOylase. In addition, the SUMO-interacting motif (SIM) is also required for efficient SUMOylation of some SUMO substrates, such as oncoprotein MTA1 15, transcriptional corepressor DAXX 16, and nucleus accumbens associated 1 (NACC1) 67. We defined a conserved SIM in the ATPase domain of MORC2 that ensures efficient MORC2 SUMOylation.

Second, dynamic SUMOylation of MORC2 is important for proper chromatin remodeling, DNA repair, and cell survival in response to DNA-damaging agents. It has been reported the importance of dynamically SUMOylated target proteins throughout cellular processes, including cell cycle progression, nuclear body assembly, proteotoxic stress, and cancer progression 68. For instance, SUMOylation and deSUMOylation of PELP1 are critical for processing of ribosomal RNA 69. Dynamically controlled SUMOylated proteins in response to DNA damage has been observed by proteomics. For example, the SUMOylated demethylase JARID1B is downregulated, whereas SUMOylation of its closely related family member JARID1C is upregulated in response to MMS 17. We observed that MORC2 SUMOylation is downregulated in response to DNA-damaging agents through attenuating the interaction of MORC2 with TRIM28. Reduction in MORC2 SUMOylation favors chromatin de-condensation that is critical for efficient DNA repair (**Figure 5**). TRIM28 phosphorylation is an early event in DDR, which enhances chromatin de-condensation during DDR by generating a motif that perturbs interactions with SIM in CHD 70. Future research should investigate whether the SIM in MORC2 is responsible for reduced interactions between MORC2 and TRIM28 during the DDR, as SIM is necessary for noncovalent interactions with SUMO. In addition, DNA damage-induced TRIM28 phosphorylation in turn represses its self-SUMOylation, leading to the de-repression of a subset cell cycle and apoptosis associated genes in response to genotoxic stresses 71-73. Interestingly, at the late stage of DNA damage, MORC2 SUMOylation restores to its basal or higher levels possibly due to the change in the interactions between MORC2 and its SUMO cycling enzymes (**Figure 5**). It has been proposed that SUMOylation resembles a molecular glue that increases protein-protein interactions 54, 74. We verified the specific interactome of the SUMOylated MORC2 in our proteomics analysis. MORC2 SUMOylation enhances its interaction with CSK21, a key regulator of DNA-PKcs activity (**Figure 6**). This observation suggests that MORC2 SUMOylation recovery at the late stage promotes DNA repair partially by recruiting DNA repair-associated kinase CSK21 to further activate DNA-PKcs. Hence, MORC2 SUMOylation stabilizes the genome and favors cancer cell survival under genotoxic stress. In support of this notion, MORC2 SUMOylation is important for DNA repair and cell survival under genotoxic stress. Reintroduction of SUMOylation-deficient MORC2 (K767R and SIM^mut1)^ into MORC2-knockout cells results in more γH2AX foci formation than cells re-expressing WT-MORC2. The growth of tumors derived from K767R mutant MORC2 cells is more strongly inhibited after ADR treatment than the growth of tumors derived from cells expressing WT MORC2 (**Figure 7**). These observations suggest that MORC2 SUMOylation facilitates DNA repair and enhances cancer cell survival in response to DNA damage.

Third, potential crosstalk among PTMs of MORC2 controls efficient DDR. Switches between SUMOylation and acetylation have been identified, such as MEF2A 75 and HIC1 76. Especially, our previous work showed that MORC2 is acetylated at K767 (K767Ac) and phosphorylated at S739 upon treatment with DNA-damaging agents, and that both PTMs are important for DNA repair 23, 30. Our results showed that MORC2 SUMOylation exhibits a crosstalk with K767 acetylation and S739 phosphorylation, thus working in concert in response to DNA damage (**Figure S7C and Figure S7D**). Future work should also examine the chronological order and interdependence of these PTMs of MORC2 in response of DNA damage, and figure out the so-called “PTM code” affecting its 3D structures and modulating its molecular functions. For instance, SUMOylation of PARP1 does not affect its ADP-ribosylation enzyme activity but completely impairs p300-mediated acetylation of PARP1, indicating an intriguing crosstalk of SUMOylation and acetylation on PARP1 77. In addition, a switch between acetylation and SUMOylation of tumor suppressor protein HIC1 (hypermethylated in cancer 1) at lysine 314 differentially regulates its transcriptional repression activity and target genes 76, 78.

In summary, findings presented here suggest that MORC2 is modified by SUMO1 and SUMO2/3 at K767, and this event is precisely regulated by SUMO E3 ligase TRIM28 and SENP1. Moreover, dynamically regulated SUMOylated MORC2 is important for chromatin remodeling and DNA repair in response to DNA damage and drives chemoresistance in breast cancer. Consequently, the use of SUMO inhibitors interfering with MORC2 SUMOylation is a potentials strategy for reversing MORC2-driven chemoresistance.

## Supplementary Material

Supplementary figures and tables.

## Abbreviations

MORC2: microrchidia family CW-type zinc finger protein 2
PTM: post-translational modification
SUMO: small ubiquitin-like modifier
SIM: SUMO interaction motif
TRIM28: tripartite motif containing 28
SENP1: SUMO specific peptidase 1
DDR: DNA damage response
CSK21: casein kinase II subunit alpha
CHD4: chromodomain-helicase-DNA-binding protein 4
DNA-PKcs: DNA-dependent protein kinase catalytic subunit
