# Supplementary material for: Dynamic SUMOylation of MORC2 orchestrates chromatin remodelling and DNA repair in response to DNA damage and drives chemoresistance in breast cancer
Source: Theranostics. 2023 Jan 22;13(3):973–90. doi: 10.7150/thno.79688 (PMC9925317; doi:10.7150/thno.79688)
Supplement: Supplementary file 1 — Supplementary figures and tables. [file thnov13p0973s1.pdf]

Supporting Information for

Zhang FL, et al. Dynamic SUMOylation of MORC2 orchestrates chromatin remodeling and DNA repair in response to DNA damage and drives chemoresistance in breast cancer

This Supplementary Information includes

Supplementary Figures S1 to S11

Supplementary Tables S1 to S4

Supplementary figures and figure legends

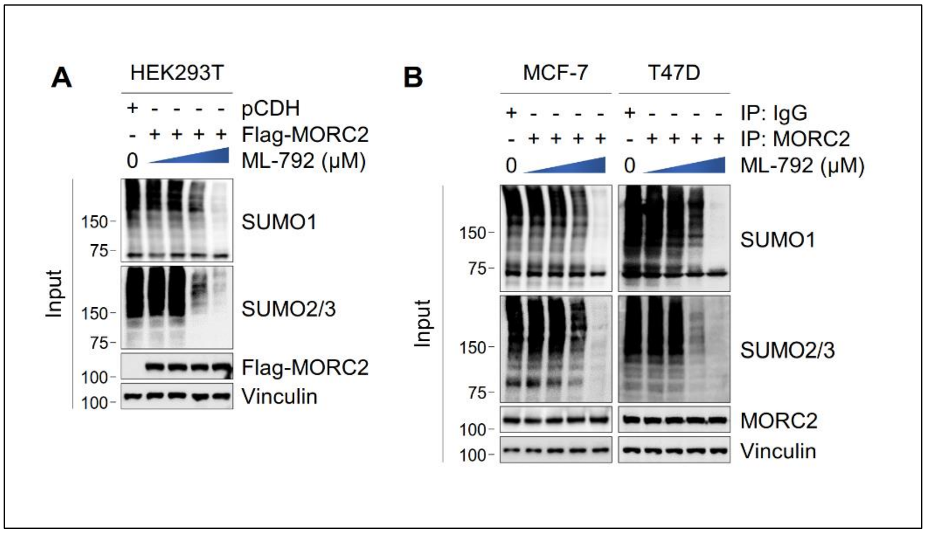

Figure S1. ML-792 efficiently inhibits MORC2 SUMOylation

(A-B) HEK293T (A), MCF-7, and T47D (B) cells were treated with increasing doses of SUMO inhibitor ML-792 (0, 0.01, 0.1 and 1 μM), and subjected to immunoblotting analysis with the indicated antibodies.

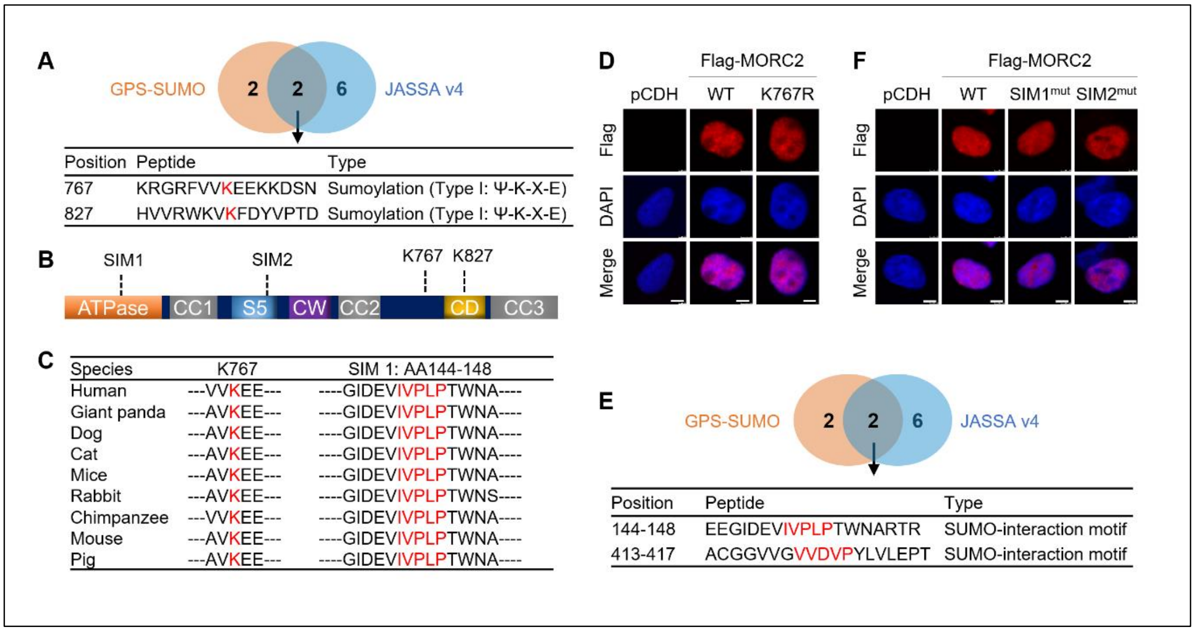

Figure S2. Prediction of SUMO modification site and SUMO-interaction motif

(A-B) Both GPS-SUMO and JASSA programs were used to predict SUMO modification sites of MORC2. Both K767 and K827 are potential SUMOylation sites of MORC2 (A), and their locations within MORC2 are shown in B.

(C) Alignment of MORC2 SUMO modification site K767 and SUMO-interaction motif SIM1 across different species.

(D) Subcellular localization of WT and K767R Flag-MORC2 were analyzed by immunofluorescent staining with an anti-Flag antibody in HEK293T cells. Nuclei were counterstained with DAPI. Scale bar: 2.5μm.

(E) GPS-SUMO and JASSA programs were used to predict SUMO-interaction motif of MORC2. Two potential SIMs (residues144-148 and 413-417) were identified (E), and their locations within MORC2 are shown in B.

(F) Subcellular localization of WT and two SIM mutant Flag-MORC2 were analyzed by immunofluorescent staining. Nuclei were counterstained with DAPI. Scale bar: 2.5μm.

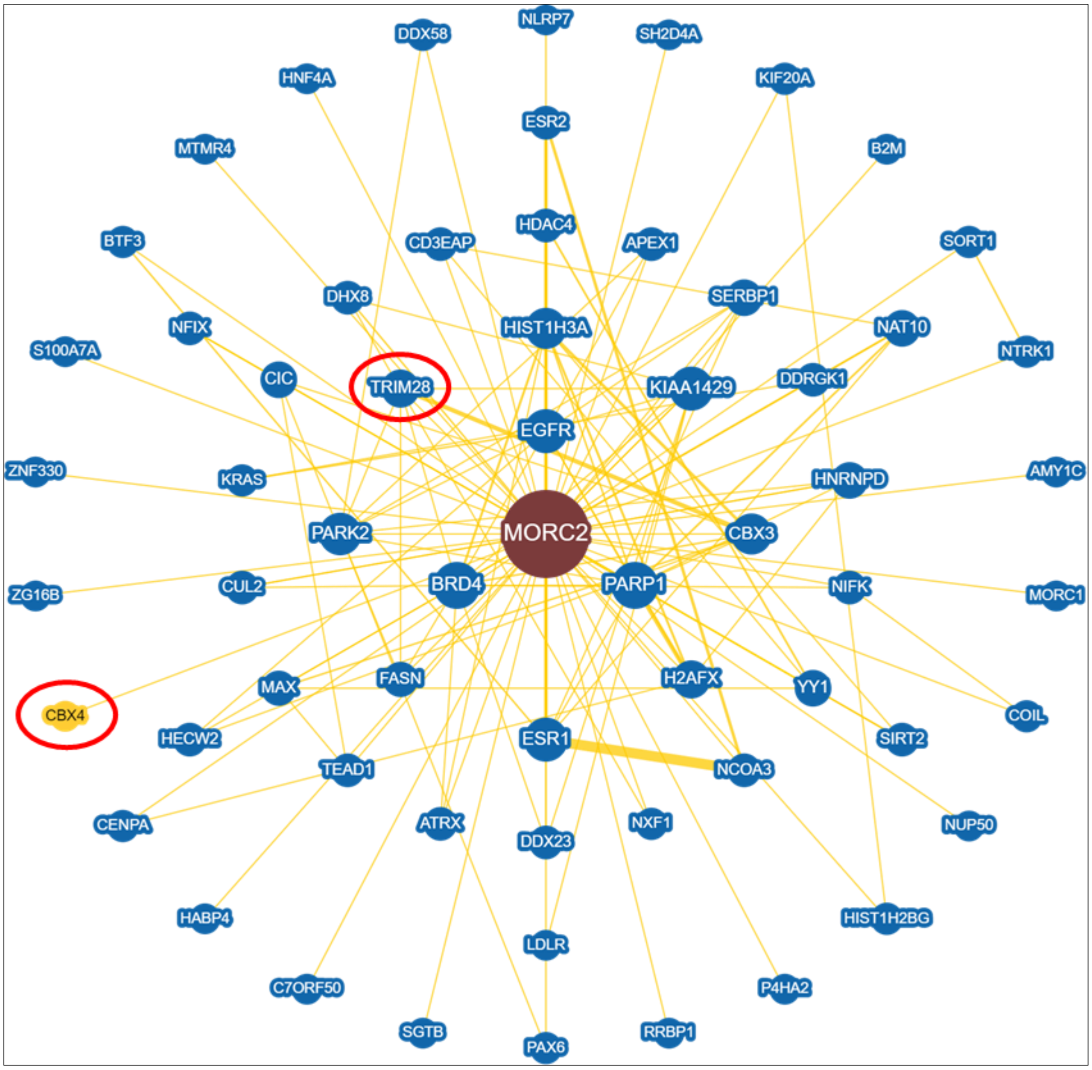

Figure S3. **MORC2-interacting proteins in database BioGRID (https://thebiogrid.org).** Two putative SUMO E3 ligases, TRIM28 and CBX4, were found as the potential binding partners of human MORC2 as indicated by red circles.

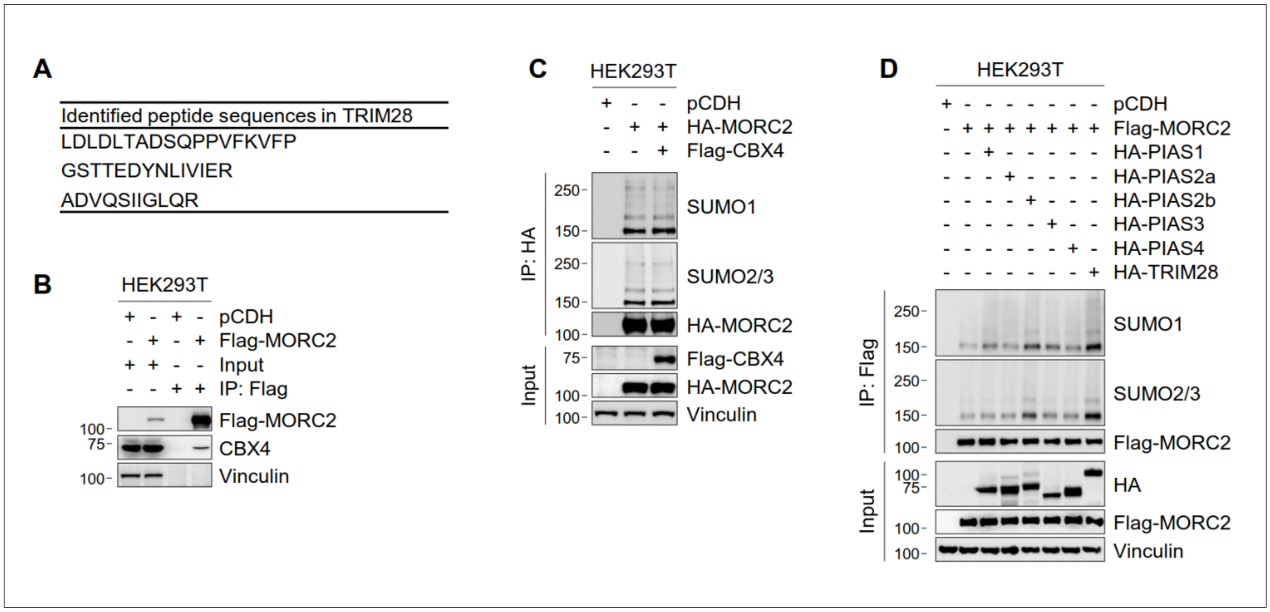

Figure S4. Screening of SUMO E3 ligases for MORC2 SUMOylation

(A) Identified sequences of TRIM28 peptides by mass spectrometry analysis of MORC2 interactome in our previous study.

(B) Flag-MORC2 was ectopically expressed in HEK293T cells, and total cellular lysates were subjected to Co-IP assays with Flag beads, followed by immunoblotting analysis with the indicated antibodies.

(C) HEK293T cells were transfected with the indicated plasmids. After 48 h of transfection, total cellular lysates were subjected to Co-IP assays with HA-beads, followed by immunoblotting analysis with the indicated antibodies.

(D) HEK293T cells were transiently transfected with Flag-MORC2 together with HA-PIAS1, HA-PIAS2a, HA-PIAS2b, HA-PIAS3, HA-PISA4 or HA-TRIM28. After 48 h of transfection, cells were lysed, and Flag-MORC2 protein was pulled down using Flag-beads, followed by immunoblotting analysis with the indicated antibodies.

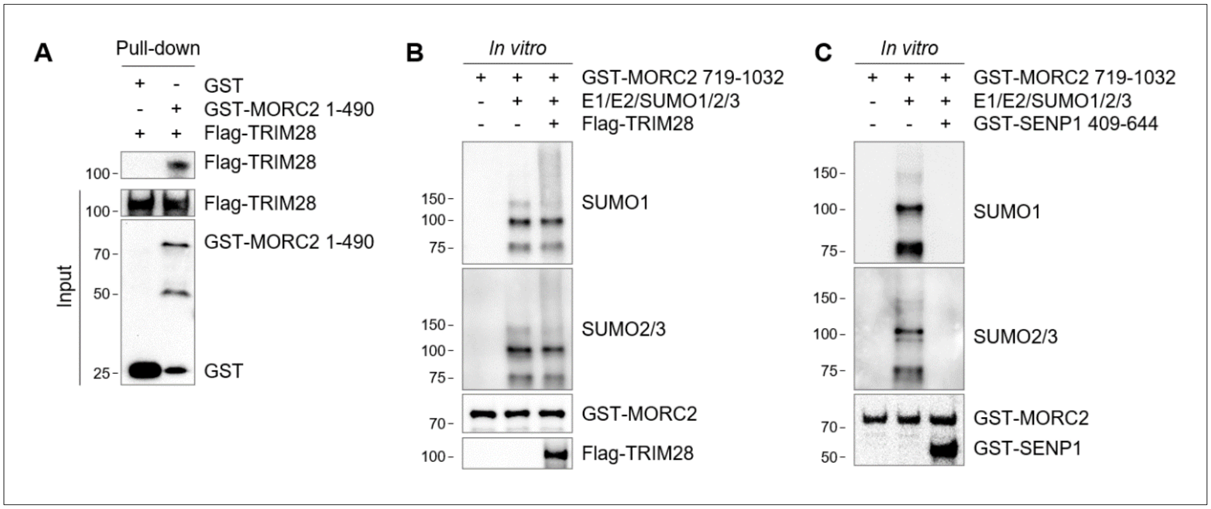

Figure S5. MORC2 SUMOylation is controlled by TRIM28 and SENP1 *in vitro*

(A) GST pull-down assays were performed using purified GST-MORC2 fragment (residues 1-490) and recombinant Flag-TRIM28. Pull down samples were subjected to immunoblotting with the indicated antibodies. GST was used as negative control.

(B) TRIM28 directly promotes the *in vitro* SUMOylation of purified GST-MORC2 fragment (residues 719-1032). *In vitro* GST-MORC2 fragment SUMOylation was enhanced in the presence of recombinant human Flag-TRIM28.

(C) SENP1 directly blocks the *in vitro* SUMOylation of purified GST-MORC2 fragment (residues 719-1032). SUMOylation of GST-MORC2 fragment was blocked when the purified active SENP1 protein fragment (residues 409-644) was added.

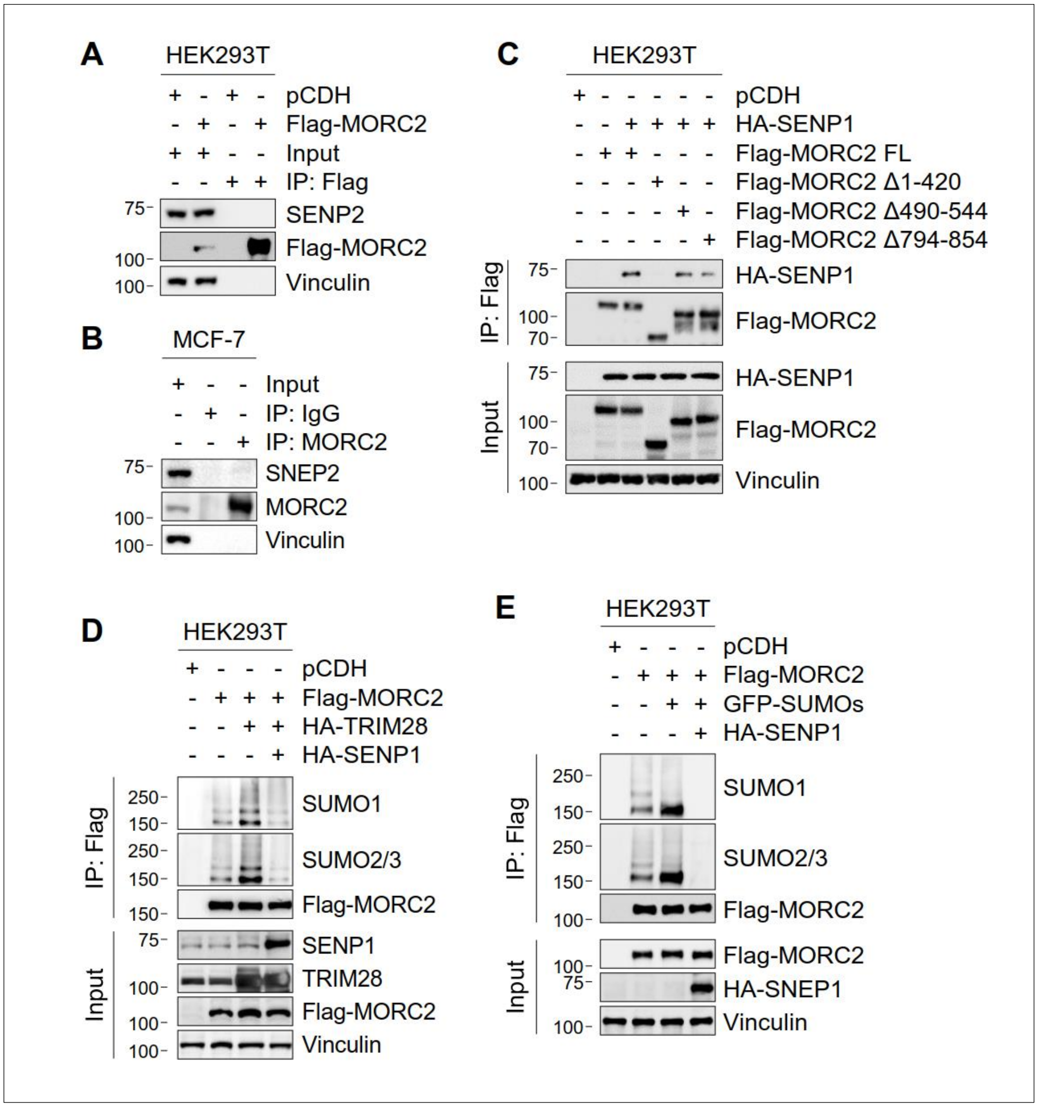

Figure S6. identification of deSUMOylating enzymes for MORC2

(A) HEK293T cells ectopically expressing Flag-MORC2 were subjected to Co-IP assays, followed by immunoblotting to detect the interaction between MORC2 and SENP2.

(B) MCF-7 cells were processed for Co-IP assays using an anti-MORC2 antibody, followed by immunoblotting with the indicated antibodies.

(C) HEK293T were transfected the indicated expression vectors, and subjected to the sequential IP and immunoblotting analyses with the indicated antibodies.

(D) HEK293T cells were transfected with Flag-MORC2, HA-TRIM28 and with or without HA-SENP1, and cell lysates were extracted and pulled down using anti-Flag beads followed by immunoblotting to detect

MORC2 SUMOylation levels.

(E) HEK293T cells were transfected with Flag-MORC2, GFP-SUMOs plasmid mix with or without HA-SENP1, and cell lysates were subjected IP and immunoblotting assays with the indicated antibodies.

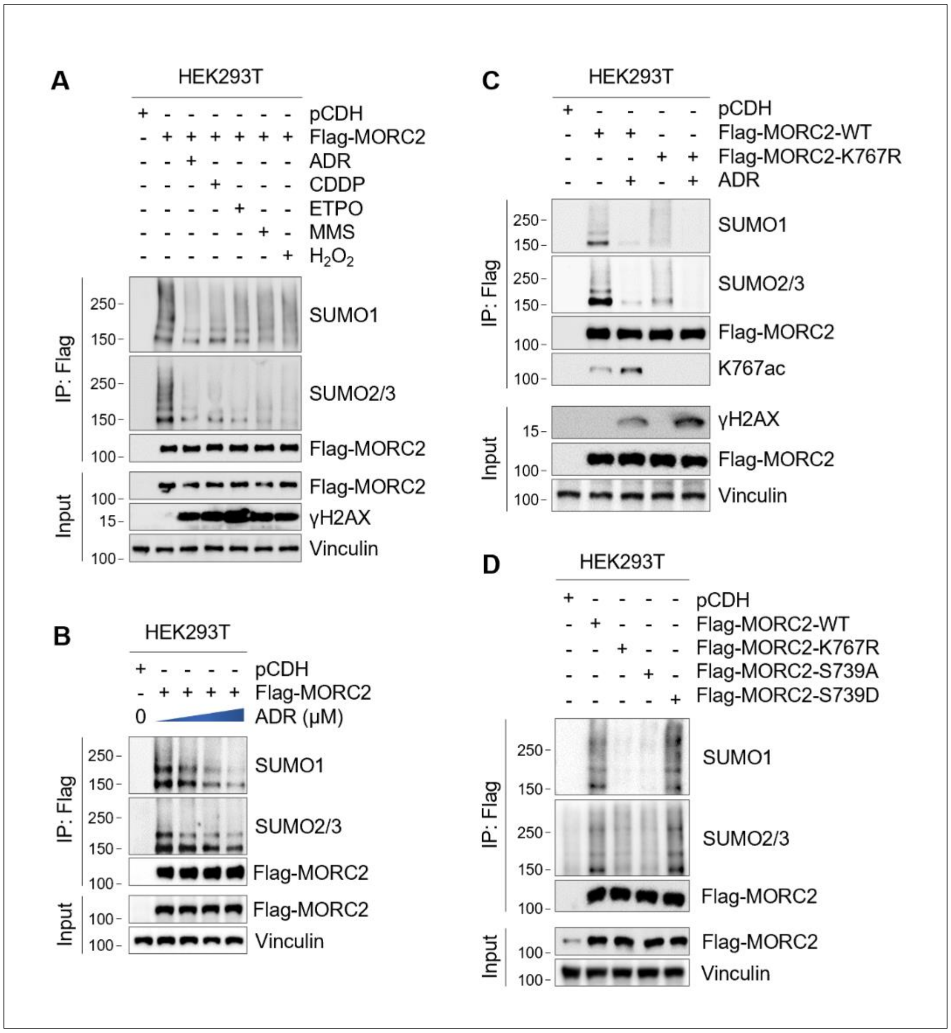

Figure S7. Transient MORC2 deSUMOylation in response to genotoxic stress

(A) HEK293T cells were transfected with Flag-MORC2 for 48 h and then treated with or without 1 μM ADR, 100 μM CDDP, 20 mM ETPO, 1 mM MMS, 1 mM H_2O_2 for 2 h. Cell lysates were processed for Co-IP assays using anti-Flag beads, followed by immunoblotting to delete the SUMOylation levels of MORC2.

(B) HEK293T cells were transfected with Flag-MORC2 for 48 h and then treated with or without increasing doses of ADR (0, 0.01, 0.1 and 1 μM) for 2 h. Cell lysates were processed for Co-IP assays using anti-Flag beads, followed by immunoblotting to delete the SUMOylation levels of MORC2.

(C) HEK293T cells were transfected with WT or K767R Flag-MORC2 for 48 h, and then treated with or without 1 μM ADR for 2 h. Cell lysates were processed for Co-IP assays using anti-Flag beads, followed by immunoblotting analyses with the indicated antibodies.

(D) HEK293T cells were transfected with Flag-MORC2 (WT, K767R, S739A or S739D) for 48 h. Total cell lysates were harvested for IP assays using anti-Flag beads, followed by immunoblotting with the indicated antibodies.

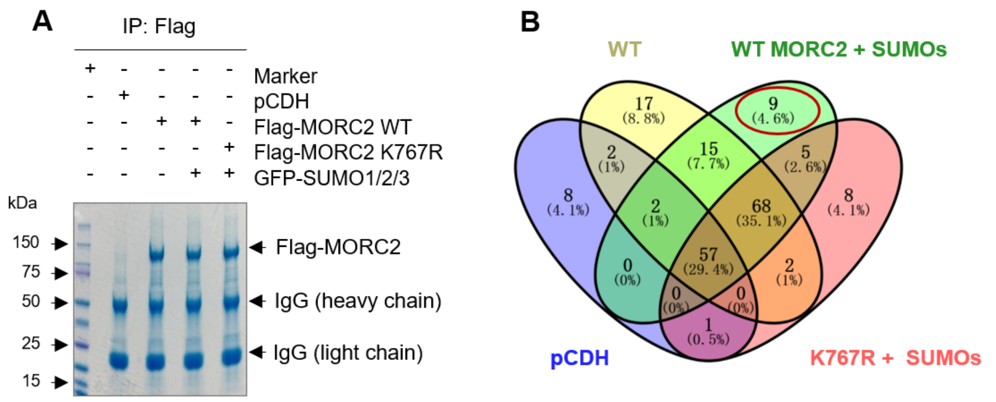

C The top 9 proteins interacting exclusively with SUMOylated WT MORC2

	Accession symbol	Full name of proteins
1	TBB4B_HUMAN	Tubulin beta-4B chain
2	SUMO1_HUMAN	Small ubiquitin-related modifier 1
3	SUMO2_HUMAN	Small ubiquitin-related modifier 2
4	DHX30_HUMAN	Putative ATP-dependent RNA helicase
5	CSK21_HUMAN	Casein kinase II subunit alpha
6	COPD_HUMAN	Coatomer subunit delta
7	NONO_HUMAN	Non-POU domain-containing octamer-binding protein
8	CHD4_HUMAN	Chromodomain-helicase-DNA-binding protein 4
9	XRCC5_HUMAN	X-ray repair cross-complementing protein

Figure S8. Analysis of binding proteins of SUMOylated MORC2

(A) HEK293T cells stably expressing control vector pCDH, WT and K767R Flag-MOTRC2 alone in combination with or without GFP-SUMO1/2/3 were subjected to Co-IP assays using anti-Flag beads followed by SDS-PAGE and Coomassie brilliant blue staining.

(B) Interacting proteins of Flag-MORC2 were identified using LC-MS/MS analysis, and the numbers of specific interacting proteins of each group are shown in Venn diagram.

(C) The nine proteins which were marked in B using red circle were exclusively identified in the SUMOylated MORC2 group. Proteins associated with DNA damage are highlighted in red.

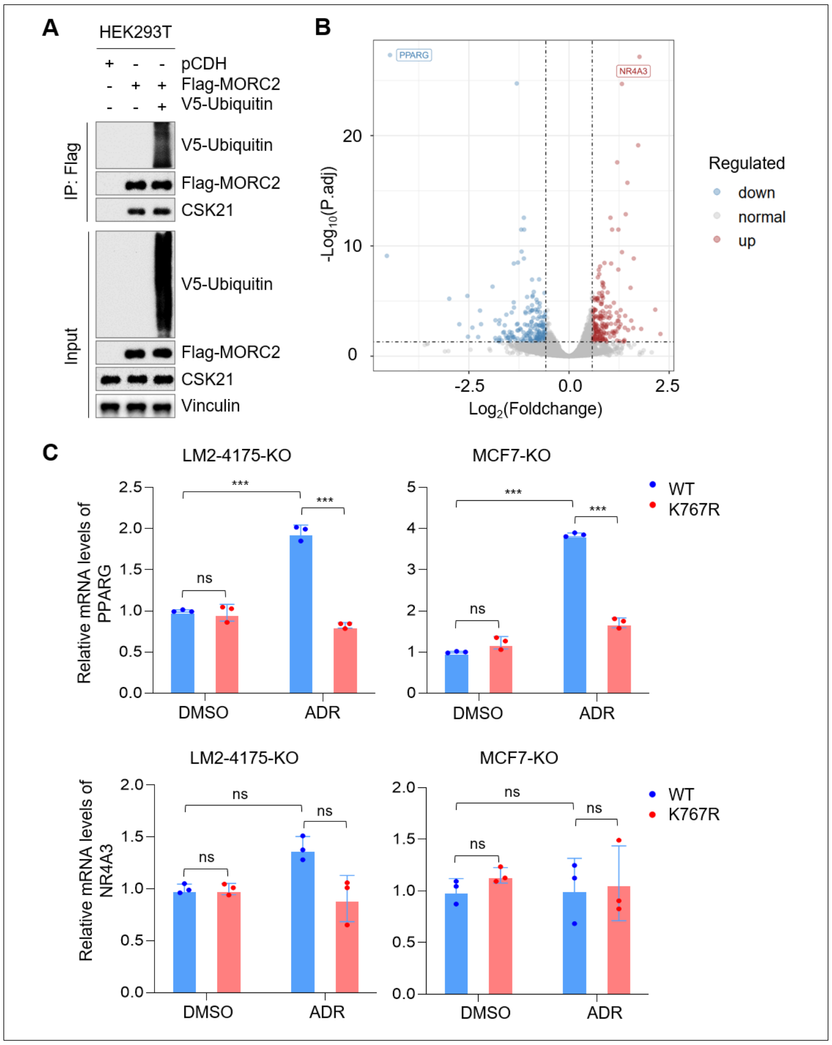

Figure S9. Analysis of binding proteins of SUMOylated MORC2

(A) HEK293T cells expressing control vector and Flag-MORC2 in combination with or without V5-ubiquitin for 48 h were subjected to Co-IP assays with anti-Flag beads, followed by immunoblotting analysis.

(B) RNA-seq data from the GEO database GSE95452 was reanalyzed. Differentially expressed genes between wild-type and MORC2 KO HeLa cells were displayed using volcano plot, and the DNA damage associated genes PPARG and NR4A3 are shown.

(C) The mRNA level for PPARG, but not NR4A3, was significantly higher in MCF7 and LM2-4175 cells expressing WT-MORC2 compared to K767R-MORC2, after treatment with ADR.

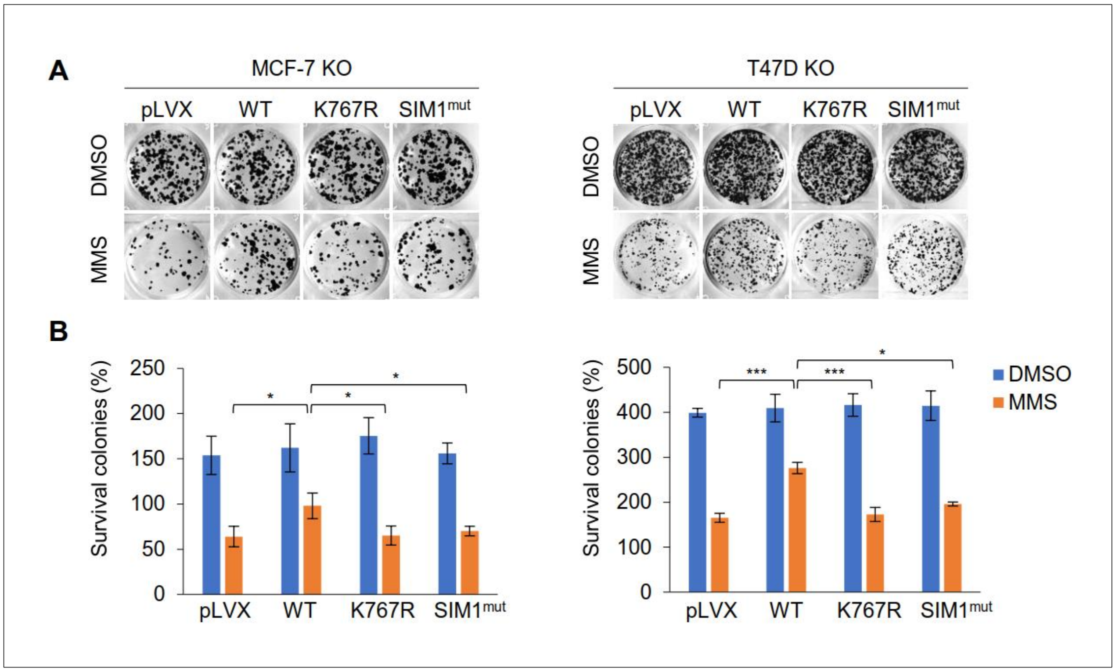

Figure S10. SUMOylated MORC2 renders breast cancer cells resistant to DNA damage agent MMS (A-B) WT, K767R and SIM1^{mut} MORC2 were reconstituted into MORC2-knockout MCF-7 and T47D cells. Cells were treated with 200 nM DNA damage agent MMS for approximately 14 days. The representative images of survival colonies are shown in A. Corresponding quantitative results (B) are represented as mean ± S.D. as indicated (n=3).

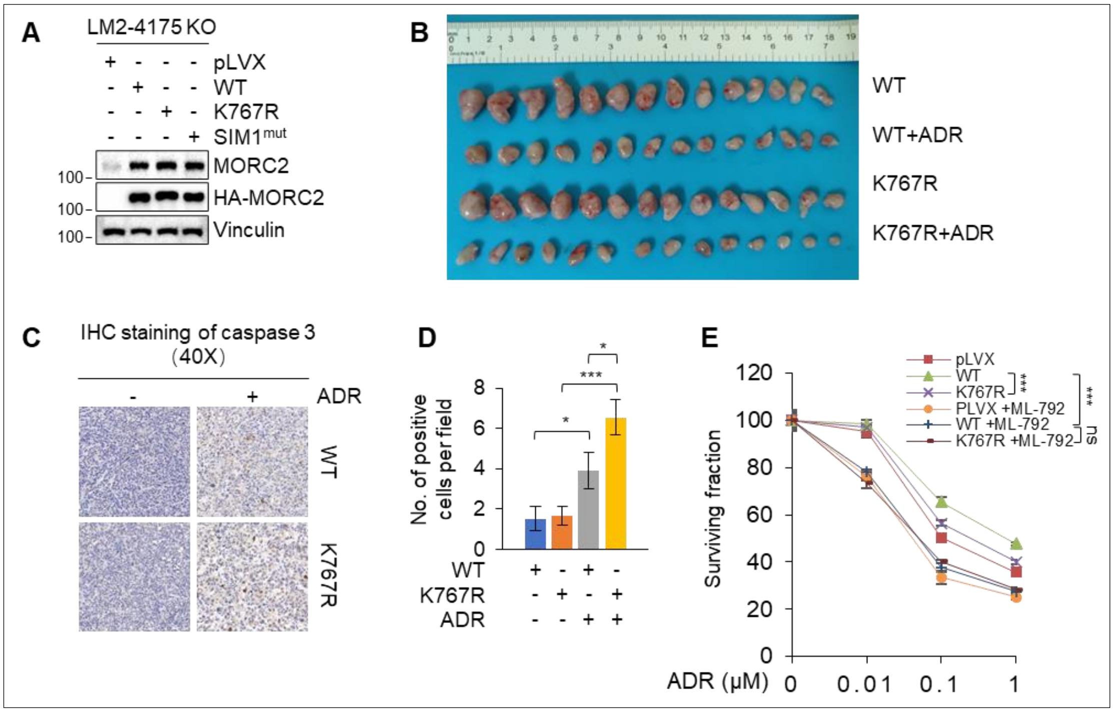

Figure S11. MORC2 SUMOylation renders breast cancer cells resistant to genotoxic drug ADR

(A) MORC2-knockout LM2-4175 cells were reconstituted with WT, K767R and $SIM1^{mut}$ HA-MORC2 through lentiviral infection and expression status were detected by Western blotting.

(B) The resultant LM2-4175 cells were injected into the subcutaneous flanks of female nude mice to establish xenograft tumors (n=14/group). ADR treatment was started when the tumor volume in one of the groups exceeded 100 mm^3. Representative images of xenograft tumors are shown.

(C-D) Representative images of IHC staining of cleaved-caspase 3. Corresponding quantitative results are shown in D.

(E) MCF-7 cells stably expressing control vector, WT or K767R MORC2 were treated with increasing doses of ADR alone or in combination with or without 0.5 μM ML-792, followed by CCK-8 assays. Quantitative results are represented as mean ± S.D. as indicated (n=3)

Supplementary Tables

Table S1. Primers used for subcloning to other vectors

Genes	Primers	Sequences
HA-MORC2	Forward	TTCCTCGAGACTAGTTCTGCCACCATGGCTTTCACAAATTAC
	Reverse	GGATCCGCGGCCGCTCTTTAAGCGTAGTCTGGGACGTCGTATGGGTAGTCCCCCTTGGTGATGA
HA-SENP1	Forward	AGAGGATCTATTTCCGGTGCCACCATGGATGATATTGCTGATAGG
	Reverse	GGCGGGATCCGCGGCCGCTTAAGCGTAGTCTGGGACGTCGTATGGGTACAAGAGTTTTCGGTGGAGG
HA-SENP2	Forward	ACCTCCATAGAAGATTCTAGAGCCACCATGTACAGATGGCTG
	Reverse	GATCCATTTAAATTCGAATTCTTAAGCGTAGTCTGGGACGTCGTATGGGTA CAGCAACTGCTGATG
HA-SENP3	Forward	ACCTCCATAGAAGATTCTAGAGCCACCATGAAAGAGACTATA
	Reverse	GATCCATTTAAATTCGAATTCTTAAGCGTAGTCTGGGACGTCGTATGGGTA CACAGTGAGTTTGCA
HA-CBX4	Forward	GGATCTATTTCCGGTGAAGCCACCATGGAGCTGCCAGCTGTTG
	Reverse	GGATCCGCGGCCGCTCTTTAAGCGTAGTCTGGGACGTCGTATGGGTACACCGTCACGTACTCCTTG
HA-UBC9	Forward	AGAGGATCTATTTCCGGTGCCACCATGTCGGGGATCGCCCTC
	Reverse	GGCGGGATCCGCGGCCGCTTAAGCGTAGTCTGGGACGTCGTATGGGTATGAGGGCGCAAACTTCTT
HA-TRIM28	Forward	AGAGGATCTATTTCCGGTGCCACCATGGCGGCCTCCGCGGCG
	Reverse	GGCGGGATCCGCGGCCGCttaAGCGTAGTCTGGGACGTCGTATGGGTAGGGGCCATCACCAGGGCC

Table S2. Primers used for cloning sgRNA and shRNA

Genes	Primers	Sequences
sgUBC9 #1	Forward	CACCGACATTCGGGTGAAATAATGG
	Reverse	AAACCCATTATTTCACCCGAATGTC
sgUBC9 #2	Forward	CACCGTATTTCCCCACAGACTCCGT
	Reverse	AAACACGGAGTCTGTGGGGAAATAC
sgSENP1 #1	Forward	CACCGGCCACAAACAGGTTTTCCAG
	Reverse	AAACCTGGAAAACCTGTTTGTGGCC
sgSENP1 #2	Forward	CACCGTGAGCCCCAAGAAAACTCAG
	Reverse	AAACCTGAGTTTTCTTGGGGCTCAC
shTRIM28 #1	Forward	CCGGCCTGGCTCTGTTCTCTGTCCTCTCGAGAGGACAG AGAACAGAGCCAGGTTTTTG
	Reverse	AATTCAAAAACCTGGCTCTGTTCTCTGTCCTCTCGAG AGGACAGAGAACAGAGCCAGG
shTRIM28 #2	Forward	CCGGGAGGACTACAACCTTATTGTTCTCGAGAACAAT AAGGTTGTAGTCCTCTTTTTG
	Reverse	AATTCAAAAAGAGGACTACAACCTTATTGTTCTCGAG AACAATAAGGTTGTAGTCCTC

Table S3. Primers used for mutation cloning and qPCR

Genes	Primers	Sequences
MORC2-K767R	Forward	GCAGATTTGTTGTGAGGGAGGAAAAGAAGG
	Reverse	CCTTCTTTTCCTCCCTCACAACAAATCTGC
MORC2-K827R	Forward	CGGTGGAAGGTGAGGTTTGACTACGTG
	Reverse	CACGTAGTCAAACCTCACCTTCCACCG
MORC2-SIM1^{mut}	Forward	AAGAAGGCATTGATGAAGTGGCAGCACCCGCGCCCACCTGGAATGCTCGG
	Reverse	CCGAGCATTCCAGGTGGGCGCGGGTGCTGCCACTTCATCAATGCCTTCTT
MORC2-SIM2^{mut}	Forward	GGTTGTTGGGGCCGCCGACGCCCCCTACCTGGTCCTGGAGCCTACACACAACAA
	Reverse	TTGTTGTGTGTAGGCTCCAGGACCAGGTAGGGGGCGTCGGCGGCCCCAACAACC
SENP1-C603S	Forward	ATGGAAGTGACAGCGGGATGTTTGC
	Reverse	GCAAACATCCCGCTGTCACTTCCAT
TRIM28-C651A	Forward	AGTGTGAGTTTTGTTTCCACCTGGACGCCCACCTGCCGGC
	Reverse	GCCGGCAGGTGGGCGTCCAGGTGGAAACAAAACTCACACT
MORC2 1-490	Forward	AGAGGATCTATTTCCGGTGCCACCATGGCTTTCACAAATTACAGC
	Reverse	GGCGGGATCCGCGGCCGCTTACTTATCGTCGTCATCCTTGTAATCAGCTCTCCGGCGTTTGTA
MORC2 491-718	Forward	AGAGGATCTATTTCCGGTGCCACCATGGCTATGGAAATCCCCACC
	Reverse	GGCGGGATCCGCGGCCGCTTACTTATCGTCGTCATCCTTGTAATCTGGAGTCTTGATGACTTTG
MORC2 791-1032	Forward	AGAGGATCTATTTCCGGTGCCACCATGGTGGTGAAGAAGACAGAG
	Reverse	GGCGGGATCCGCGGCCGCTTACTTATCGTCGTCATCCTTGTAATCGTCCCCCTTGGTGATGAG
PPARG qPCR	Forward	GGGATCAGCTCCGTGGATCT
	Reverse	TGCACTTTGGTACTCTTGAAGTT
NR4A3 qPCR	Forward	TGCGTCCAAGCCCAATATAGC
	Reverse	GGTGTATTCCGAGCTGTATGTCT

Table S4. Information for primary antibodies used in this study

Antibodies	Vendors	Cat#	Species	WB	IHC	IP	IF
SUMO1	Abcam	ab32058	Rabbit monoclonal	√	√	√	√
SUMO2/3	Abcam	ab81371	Mouse monoclonal	√	√	√	√
MORC2	Bethyl	A300-149	Rabbit polyclonal	√		√	
Vinculin	Sigma	V9131	Mouse monoclonal	√			
Flag	Sigma	F3165	Mouse monoclonal	√		√	
GFP	Abcam	ab32146	Rabbit monoclonal	√			
HA	CST	C29F4	Rabbit polyclonal	√		√	
UBC9	Abcam	ab75854	Rabbit monoclonal	√	√	√	
SENP1	Abcam	ab108981	Rabbit monoclonal	√	√		√
SENP2	Abcam	ab124724	Rabbit monoclonal	√	√		√
SENP3	Abcam	ab124790	Rabbit monoclonal	√			
CBX4	Abcam	ab174300	Rabbit monoclonal	√			
TRIM28	Abcam	ab109287	Rabbit monoclonal	√	√		√
H3K9me3	CST	13969T	Rabbit monoclonal	√	√	√	√
γH2AX	Abcam	ab81299	Rabbit monoclonal	√	√	√	√
CHD4	Abcam	ab181370	Rabbit monoclonal	√			√
XRCC5	Abcam	ab80592	Rabbit monoclonal	√	√	√	√
DNA-PKcs	Abcam	ab32566	Rabbit monoclonal	√	√		
Phospho-DNA-PKcs	Abcam	ab124918	Rabbit monoclonal	√	√		√
CSK21	Abclonal	A1014	Rabbit polyclonal	√	√		√
GST	CST	2625T	Rabbit monoclonal	√		√	
